# Modulating STDP Balance Impacts the Dendritic Mosaic

**DOI:** 10.3389/fncom.2017.00042

**Published:** 2017-06-09

**Authors:** Nicolangelo Iannella, Thomas Launey

**Affiliations:** ^1^School of Mathematical Sciences, University of NottinghamNottingham, United Kingdom; ^2^Computational and Theoretical Neuroscience Laboratory, Institute for Telecommunications Research, University of South AustraliaMawson Lakes, SA, Australia; ^3^Laboratory for Synaptic Molecules of Memory Persistence, RIKEN, Brain Science InstituteSaitama, Japan

**Keywords:** STDP balance, dendritic efficacy mosaic, functional compartments, dendritic spike generation, mutual information index

## Abstract

The ability for cortical neurons to adapt their input/output characteristics and information processing capabilities ultimately relies on the interplay between synaptic plasticity, synapse location, and the nonlinear properties of the dendrite. Collectively, they shape both the strengths and spatial arrangements of convergent afferent inputs to neuronal dendrites. Recent experimental and theoretical studies support a clustered plasticity model, a view that synaptic plasticity promotes the formation of clusters or hotspots of synapses sharing similar properties. We have previously shown that spike timing-dependent plasticity (STDP) can lead to synaptic efficacies being arranged into spatially segregated clusters. This effectively partitions the dendritic tree into a tessellated imprint which we have called a dendritic mosaic. Here, using a biophysically detailed neuron model of a reconstructed layer 2/3 pyramidal cell and STDP learning, we investigated the impact of altered STDP balance on forming such a spatial organization. We show that cluster formation and extend depend on several factors, including the balance between potentiation and depression, the afferents' mean firing rate and crucially on the dendritic morphology. We find that STDP balance has an important role to play for this emergent mode of spatial organization since any imbalances lead to severe degradation- and in some case even destruction- of the mosaic. Our model suggests that, over a broad range of of STDP parameters, synaptic plasticity shapes the spatial arrangement of synapses, favoring the formation of clustered efficacy engrams.

## Introduction

Activity-dependent changes in the firing properties of cortical neurons can arise from modifying the spatial arrangement of afferent fibers converging onto dendrites and their corresponding synaptic strengths (Poirazi et al., 2003a; De Roo et al., 2008; McBride et al., 2008). The pattern of activity conveyed by such afferents can either strengthen (Bliss and Gardner-Medwin, 1973; Bliss and Lomo, 1973) or weaken (Kirkwood and Bear, 1994) stimulated synapses. Such physiological changes are believed to represent a substrate for learning and memory; however the mechanisms responsible for the spatial arrangement have yet to be fully elucidated.

Experiments show that synaptic plasticity exhibits both associativity (McNaughton et al., 1978; Levy and Steward, 1979) and cooperativity (McNaughton et al., 1978) between synapses; where groups of stimulated synapses can collectively induce either LTP or LTD but are each individually incapable of inducing change. Experiments have also identified two additional properties. The first is heterogeneity (nonuniformity) in the form, induction and location of expression of different types LTP and LTD. The second is temporal specificity where the temporal order and separation of pre-synaptic and post-synaptic firing determines whether a synapse is potentiated or depressed. The magnitude of change is characterized by a temporally asymmetric function of spike timing that describes a “critical window” for such alterations (Markram et al., 1997b; Bi and Poo, 1998; Debanne et al., 1994, 1998; Zhang et al., 1998).

This latter form of plasticity is typically called spike timing-dependent plasticity (STDP). The discovery of STDP has stimulated many experimental and theoretical studies on the role of action potential timing with respect to the development of cortical circuits. In particular, previous theoretical studies have typically used formulations of STDP that allows it to behave as a competitive learning rule (even though the weakly competitive version of multiplicative STDP has also been used), illustrating that the temporal asymmetric window allows the neuron to learn some temporal structure of its input, even under noisy conditions (Song et al., 2000; Song and Abbott, 2001; Gutig et al., 2003).

Historically, competitive learning rules have been important in explaining not just learning the temporal structure embedded in the afferent inputs to target neurons, but also the formation of the various types of cortical maps, most notably the development of orientation and ocular dominance columns (Kohonen, 1982; Tanaka, 1990; Miyashita and Tanaka, 1992; Miller, 1994; Erwin et al., 1995; Swindale, 1996; Young et al., 2007). This suggests potential link between competitive learning, functional map formation, and the segregation of independent input streams onto dendrites. This link has been recently discussed in Narayanan and Johnston (2012) where the authors have argued that various functional maps can be imprinted onto dendrites, each serving different roles.

There is growing interest in the nonlinear synergy between dendritic excitability and synaptic plasticity in spatially extended neuron models (Mel, 1992a; Zador et al., 1992; Poirazi et al., 2001) or STDP (Rumsey and Abbott, 2004, 2006; Iannella and Tanaka, 2006; Rabinowitch and Segev, 2006a,b; Gidon and Segev, 2009; Iannella et al., 2010). Notably, our previous studies have demonstrated that the synaptic strengths of axons from different functional streams of inputs organize themselves into spatially segregated clusters. This emergent property relies on an STDP rule admitting strong competition between synapses (Iannella and Tanaka, 2006; Iannella et al., 2010).

Here, we investigate these effects using spike timing-dependent plasticity (STDP) in a biophysically detailed model of a reconstructed layer 2/3 pyramidal cell. In this model, the neuron receives inputs independently from multiple yet equally sized groups of correlated fibers. We focus on the role of STDP (im)balance in altering the spatial representation of synapses in dendrites and especially in the emergence of spatially segregated clusters of synapse with similar properties and representing the different input streams. We conclude that dendritic mosaic robustly emerge over a wide range of dendrite morphology, mean input frequencies and degree of balance, in a nonlinear and unpredictable manner.

## Materials and methods

### Assessing differences in spatial patterns

There are measures that can quantify the spatial differences or dissimilarity between the resulting spatial organization of synaptic efficacy from two respective groups for various levels of competition. One such measure is called the spatial dissimilarity index (SDI) (Duncan and Duncan, 1955; Traulsen and Claussen, 2004). This index measures of the dissimilarity or equivalently the overlap between two spatial patterns, where segregated or similar patterns give a value close to unity or zero, respectively. The SDI is formulated as:

(1) $$\text{SDI}=\frac{1}{2}\sum_{\text{j}}\left|\frac{W_{\text{j}}^{\text{A}}}{W_{\text{tot}}^{\text{A}}}-\frac{W_{\text{j}}^{\text{B}}}{W_{\text{tot}}^{\text{B}}}\right|,$$

where $W_{\text{j}}^{\text{A},\text{B}}$ are the total synaptic efficacies at dendritic position j contributed by groups A and B, respectively, and $W_{\text{tot}}^{\text{A},\text{B}}$ are the total synaptic efficacies summed over all dendritic sites for each of these two groups.

To assess the spatial differences between multiple (more than two) spatial patterns, the above described index is replaced by one based upon mutual information. We have previously used the multigroup mutual information index (mMHI) (Iannella et al., 2010), defined as

(2) $$\text{mMHI}=\sum_{j}\frac{W_{\cdot \text{j}}}{W_{\text{tot}}}\sum_{m}\pi_{\text{jm}}\ln\left(\frac{\pi_{\text{jm}}}{\pi_{\text{m}}}\right),$$

where subscripts *j* denotes dendritic location and *m* indexes the particular afferent group where:

$$\begin{array}{l} W_{\cdot j}=\sum_{m}W_{mj}\text{ total synaptic efficacy at dendritic location }j.\\ W_{m\cdot }=\sum_{j}W_{mj}\text{ total of group }m\text{'s synaptic efficacies.}\\ W_{tot}=\sum_{m,j}W_{mj}\text{ total synaptic efficacy contributed by all groups}.\\ \pi_{m}=\frac{W_{m\cdot }}{W_{tot}}\text{ proportion of group }m\text{ synaptic weights.}\\ \pi_{jm}=\frac{W_{mj}}{W_{\cdot j}}\text{ proportion of group }m\text{ synaptic weights at }j\text{.} \end{array}$$

To quantify the degree of spatial segregation between multiple spatial patterns.

### The layer 2/3 pyramidal cell model

A biophysically detailed compartmental model of a reconstructed layer 2/3 pyramidal neuron receiving randomly timed excitatory and inhibitory synaptic inputs across the dendrite, was simulated using the NEURON simulation package (Hines and Carnevale, 2001). The model consisted of 119 sections with 263 segments, including a simplified myelinated axon, similar to those used in previous studies (Mainen et al., 1995; Iannella and Tanaka, 2006), consisting of a hillock, initial segment, five nodes and five myelin internodes, respectively. The parameters and channel types used in the simplified axon were the same as those used in Iannella and Tanaka (2006). A variety of synaptic receptors, voltage and calcium dependent ion channels known to exist in real layer 2/3 pyramidal cells were incorporated into the model. These receptors included the α-Amino-3-hydroxy-5-methyl-4-isoxazolepropionic acid (AMPA) receptor, the calcium permeable N-methyl-D-aspartate (NMDA) receptor, the ionotropic and G-protein coupled gamma-aminobutyric acid receptors (GABA_A_ and GABA_B_), respectively. The included ion channel currents were a passive leak *I*_leak_, the fast sodium *I*_Na_, the delayed rectifier potassium *I*_Kdr_, the hyperpolarization activated potassium *I*_h_, the transient A-type potassium *I*_A_, a muscarinic potassium *I*_M_, the T-type calcium *I*_T_, (high voltage activated) L-type calcium *I*_HVA_, the calcium dependent potassium C-type *I*_C_, the medium after hyperpolarization (AHP) *I*_mAHP_; and the slow AHP *I*_sAHP_. These channels were distributed throughout the dendrites, soma, and axon with densities according to published experimental data from the rat. In case when data was absent, both distributions and parameter values similar to those used in previous studies were used. Finally, passive properties used in our Layer 2/3 model neuron, were similar or adopted from previous investigations (Mainen et al., 1995; Iannella et al., 2004, 2010): the dendritic membrane capacitance was *C*_*m*_ = 0.9 μF/cm^2^, the resting potential was −80 mV, and the internal resistivity R_a_ was 200 Ωm.

A full description of the ion channels, their corresponding currents and distributions used in the simulations were similar or identical to those used in previous modeling studies (Rhodes and Gray, 1994; Mainen et al., 1995; Rhodes and Llinás, 2001; Traub et al., 2003; Iannella et al., 2004, 2010; Iannella and Tanaka, 2006). These descriptions are detailed in the Supplementary Materials.

Stimulation to the Layer 2/3 pyramidal cell was provided by a single inhibitory group consisting of 250 afferent fibers and either two of four groups of equally sized groups of correlated excitatory fibers. Here, when two groups were used there were 500 afferent fibers per excitatory group, while for stimulation originating from four groups there were 250 afferents per group. Furthermore, there were no or little correlation between the activity carried by any single afferent fiber from one group and those from any other group. Put simply, the activity within any single group was correlated, but the activity of afferents between different groups were not correlated with each other. Finally, the activity carried by excitatory and inhibitory fibers are also uncorrelated. These excitatory groups will be labeled alphabetically, i.e. for two groups they will be referred to as groups A and B, while for four, they will referred to as groups A, B, C, and D. Whether inhibitory or excitatory, each afferent fiber forms five synaptic contacts at randomly chosen locations throughout the dendrite of the model, as suggested by current anatomical data (Thomson et al., 1994, 2002; Markram et al., 1997a; Feldmeyer et al., 2002).

Each simulation began by allowing each excitatory afferent fiber to connect to five randomly selected positions across the dendrite. Similarly, each inhibitory afferent also made five synapses at locations randomly selected throughout the initial segment, hillock, soma, and dendrite. All synapses were activated at random times. Inhibitory fiber activity was modeled via a temporally homogeneous Poisson processes with a mean frequency of 10 Hz. Excitatory afferent activity was modeled as correlated Poisson processes where the activity of a particular group contains higher order statistics (correlations) (Kuhn et al., 2003). These correlations are mediated by coincident activity involving distinct subsets of fibers that only belong to a single group of afferents, while there is no correlation between the activity of any pair of input fibers that belong to different groups, i.e., the cross-correlation between these fibers is zero. The adopted within group correlation coefficient was set to *C* = 0.05, with a mean firing rate for all excitatory fibers of 40 Hz, accepted where otherwise stated.

### STDP learning rule

The synaptic weights of AMPA conductances *w*_j_(*t*) ∈ [0, 1] were altered by STDP, (NMDA, GABA_A_ and GABA_B_ conductances remained fixed). Learning was implemented using a nonlinear STDP rule (Gutig et al., 2003). For clarity, this rule is described below.

#### Gutig rule: pair based nonlinear STDP

(3) $$\Delta w_{\text{j}}=\{\begin{array}{ll} A_{+}{\left(1-w_{\text{j}}\right)}^{\mu }\exp\left(-|\Delta t|/\tau_{+}\right) & \text{if}\Delta t>0\\ -A_{-}w_{\text{j}}^{\mu }\exp\left(-|\Delta t|/\tau_{-}\right) & \text{if}\Delta t\leq 0 \end{array}$$

where Δ*t* = *t*^post^ − *t*^pre^ denotes the timing difference between pre-synaptic and post-synaptic events. *A*_+_ and *A*_−_ are positive constants scaling the magnitude of individual weight changes, and τ_+_ and τ_−_ are time constants determining the size of the temporal learning window in which potentiation and depression occurs. The pre-synaptic event *t*^pre^ denotes the arrival time of pre-synaptic input to some specific dendritic location, while the post-synaptic event *t*^post^ typically denotes the time when a local dendritic spike was generated. When Δ*t* is positive, synaptic efficacy is potentiated, and depressed otherwise; where individual changes in synaptic efficacy *w*_j_ are also weight dependent. This weight dependence has the form of a power law where the exponent μ is a positive constant. This STDP rule has a nonlinear weight dependence when changing the weights of AMPA receptors. One can't help to notice that the additive STDP rule is recovered when μ = 0 and corresponds to changes in synaptic efficacy that are independent of the weight *w*_j_; while the multiplicative rule is recovered when μ = 1, corresponding to linearly dependent weight changes. The parameters used for potentiation and depression components of this learning rule were *A*_+_ = 0.0025, *A*_−_ = 0.001125, τ_+_ = 13.5 ms and τ_−_ = 34.5 ms, in agreement with previous experimental observations (Froemke and Dan, 2002; Froemke et al., 2005). Detection of post-synaptic events were recorded when the local membrane potential surpasses a pre-specified threshold of θ = −20 mV.

## Results

### Competition dependent emergence of clustered synaptic efficacy engrams

The formation of spatial patterns displaying a clustered organization typically emerge by balancing the requirements of co-operation and competition of some limited resource. In the case of Gütig's nonlinear STDP (see Materials and Methods—STDP learning rules), synapses compete both spatially and temporally to control the timing of somatic and/or dendritic spike generation. This competition is believed to take the form of a spatio-temporal winner-take-all process that ultimately leads to the formation of synaptic efficacy clusters. A key feature of Gütig's nonlinear STDP rule is the presence of the exponent μ. This parameter controls the weight dependence of the rule and can be interpreted as a parameter that controls the degree of competition, since μ = 0 corresponds to the additive STDP and exhibits strong competition (Song et al., 2000; Song and Abbott, 2001); while μ = 1 recovers the multiplicative STDP rule, a rule known to display stable yet weak competition dynamics (van Rossum et al., 2000). For intermediate values of μ the weight dependence is nonlinear and can be interpreted as introducing some intermediate degree of strong and weak competition.

To assess spatial segregation and complementarity in the case of two independent stream of inputs, we used the spatial dissimilarity index (SDI) (Duncan and Duncan, 1955; Traulsen and Claussen, 2004) (see Section Materials and Methods). Note that two spatial patterns are considered to be complementary when they possess a reciprocal relationship in space so that one pattern can be considered the negative image of the other. This index provides a quantitative measure of the difference between two spatial patterns. Two patterns are similar if the SDI is close to zero, while values close to unity indicates that two patterns are spatially segregated.

**Figure 1:**
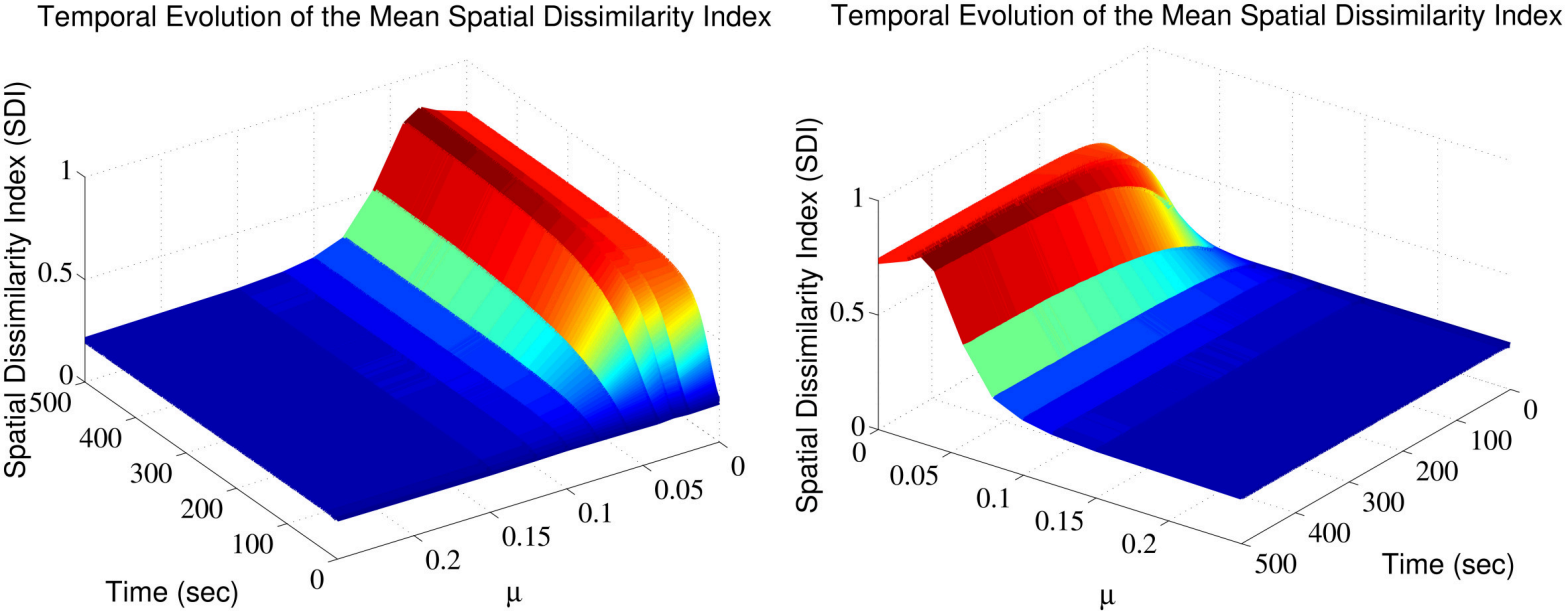
Synaptic efficacy clusters and spatial complementarity depends upon synaptic competition. The mean SDI calculated over 10 randomly initialized trials, as a function of both μ and time. We jointly observe the temporal evolution and the effect of altering competition on the resulting SDI.

The SDI varies over time, in a manner that depends on the exponent μ (Figure 1). Note that for each trial, a different random seed was used to initialize the simulation, assuring that both the pattern of afferent connectivity and the activity seen at any location in the dendrite are different between trials. There is an optimum value for μ maximizing the SDI; for which synaptic competition produced the largest degree of spatial complementarity. Above the optimum, high value of μ weakens synaptic competition, leading to an increase in the average size of the efficacy engram contributed by each respective group of excitatory fibers. Such increases in size increases the overlap between the clusters contributed by each respective group of excitatory afferent fibers, to the point where there is little difference between them. More surprisingly, too high competition also leads to cluster fragmentation and SDI decrease.

### STDP balance modulates spatial segregation and complementarity

Another important facet is to determine how altering the balance between potentiation and depression (*A*_−_τ_−_/*A*_+_τ_+_ ratio), impacts the formation of clustered synaptic efficacy engrams. Previous theoretical studies, using the simple integrate-and-fire model neuron, have shown that in order to avoid unphysiological increase of synaptic weights, this ratio is important for stable yet competitive learning (Song et al., 2000; Song and Abbott, 2001). However, for a spatial model neuron, the impact of such alterations was unknown. This issue was directly addressed here by increasing the maximal amplitude of the STDP learning window's depression component *A*_−_. Again SDI was averaged from ten trials, each initialized using a different random seed.

The imbalance between depression and potentiation in STDP was increased from 1 to 6, for which synaptic depression is overwhelmingly dominant and results essentially in silencing the neuron. Furthermore, this avoided the situation where, irrespective of the afferent group, the total synaptic efficacy contributed by a single group at any location was zero, i.e., $W_{\text{tot}}^{\text{A}}=0$ and/or $W_{\text{tot}}^{\text{B}}=0$. Beyond these conditions (ratios > 6), numerical singularities prevented SDI calculation.

**Figure 2:**
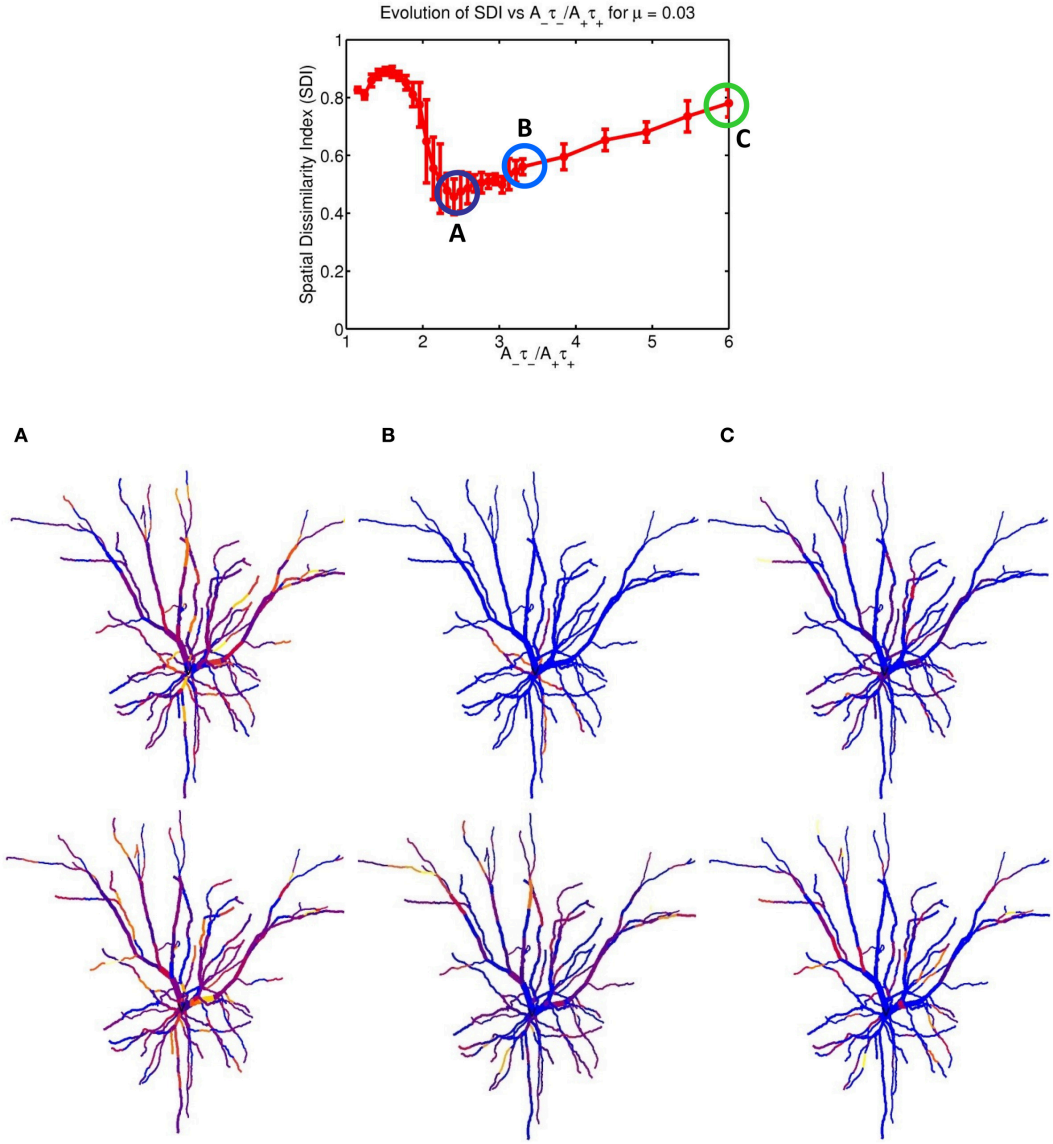
STDP balance controls efficacy cluster formation. Each data point of the mean SDI was calculated using 10 randomly initialized trials, as a function of STDP balance ratio *A*_−_τ_−_/*A*_+_τ_+_. The balance was changed by systematically incrementing *A*_−_ by 0.0001 until a balance value of 3.3 then by 0.005, up to a ratio of 6. Increasing the imbalance between depression and potentiation leads to initial degradation in the SDI, and further increases leads to a recovery of clustering. The spatial distribution of clusters is illustrated for the three balance ratio values of 2.25, 3.3 and 6 (column denoted by **A–C**, respectively), for each specific afferent group. The color coding indicate the synaptic strength for the two afferent group, for each compartment. For a ratio of 2.25 (lowest SDI) each branch receive essentially similar input strength from the two afferent groups while **(B,C)** show increasing segregation.

**Figure 3:**
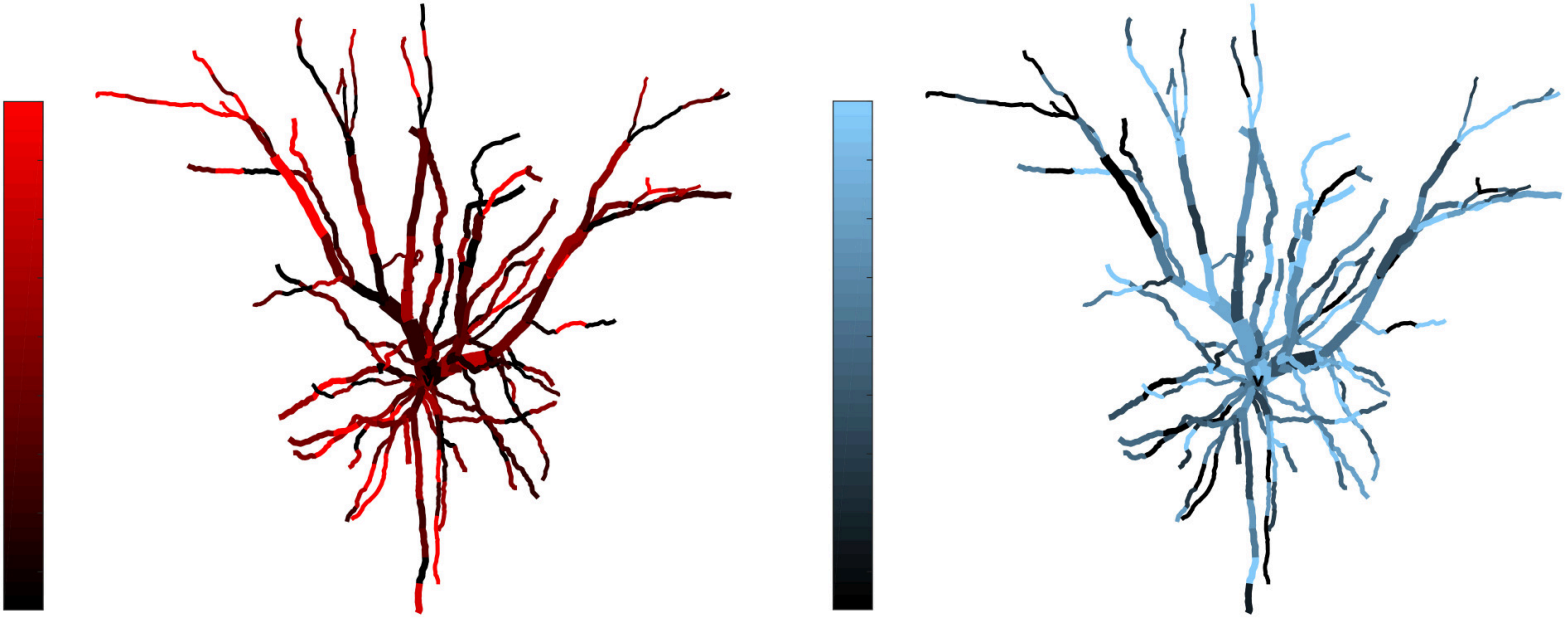
Example of complementarity. Spatial distribution of synaptic efficacy after STDP learning for μ = 0.03 and a balance ratio of 1.5 (the maximum of the SDI plot in Figure 2). The color coding represents the normalized weights for a each afferent group. Note that the patterns complement each other, with one afferent being strong where the other is weak, thus giving rise to a large SDI.

Figure 2 indicates that increasing the imbalance from 1.05 to 6 resulted in a strong non-monotonous effect on the emergence of synaptic clusters across the dendrite. Firstly, there is a clear maximum SDI value indicating that for that particular value of the ratio, the degree of spatial complementarity between the two patterns is greatest. Secondly, increasing the ratio to a value of 2.25 eventually leads to a minimum SDI value of ≈ 0.45, with no clear segregation of synaptic clusters. Finally, further increases in the balance ratio *A*_−_τ_−_/*A*_+_τ_+_ to 3.3 (Figure 2B) and to 6 (Figure 2C) leads to increased spatial segregation of synaptic efficacies across the dendrite, contributed by each afferent group (appearing in Figures 2B,C), and gives rise to a surprising recovery in the value of the SDI, where spatially segregated efficacy clusters (appearing to the right of the central plot) are still present in the dendrite. Intuitively, our expectation was that increasing the imbalance by favoring LTD, would lead to degradation of the clustering; surprisingly, the synaptic efficacy clusters were still present but at the cost of decreased spatial complementarity of the original tiling pattern. Here, complementarity is referred as the amount of spatial segregation and overlap between two patterns where one pattern can viewed as the “negative” of the other, and example is provided in Figure 3 for an imbalance ratio of 1.5 where one group dominates regions in dendritic space where the second group does not. Interestingly, a very steep transition separates the SDI maxima and minima, with an inflection point at 2. While the exact position of the inflection point is irrelevant (as it is probably multi-factorial), this reveals a high sensitivity of the model to LTP/LTD balance that may be detected experimentally.

### STDP balance impacts the formation of the dendritic mosaic

We have observed the formation of synaptic efficacy clusters when there are only two different groups of afferent axons. When more than two streams of inputs are simulated, altering the balance ratio *A*_−_τ_−_/*A*_+_τ_+_ admitted by the STDP rule also leads to changes in the formation of dendritic mosaic. To quantify clustering in these multi stream conditions, we have previously identified the multi-group Mutual Information Index (mMHI) to be a suitable metric (Iannella et al., 2010; see Materials and Methods section for details).

(4) $$\text{mMHI}=\sum_{j}\frac{W_{\cdot \text{j}}}{W_{\text{tot}}}\sum_{m}\pi_{\text{jm}}\ln\left(\frac{\pi_{\text{jm}}}{\pi_{\text{m}}}\right),$$

**Figure 4:**
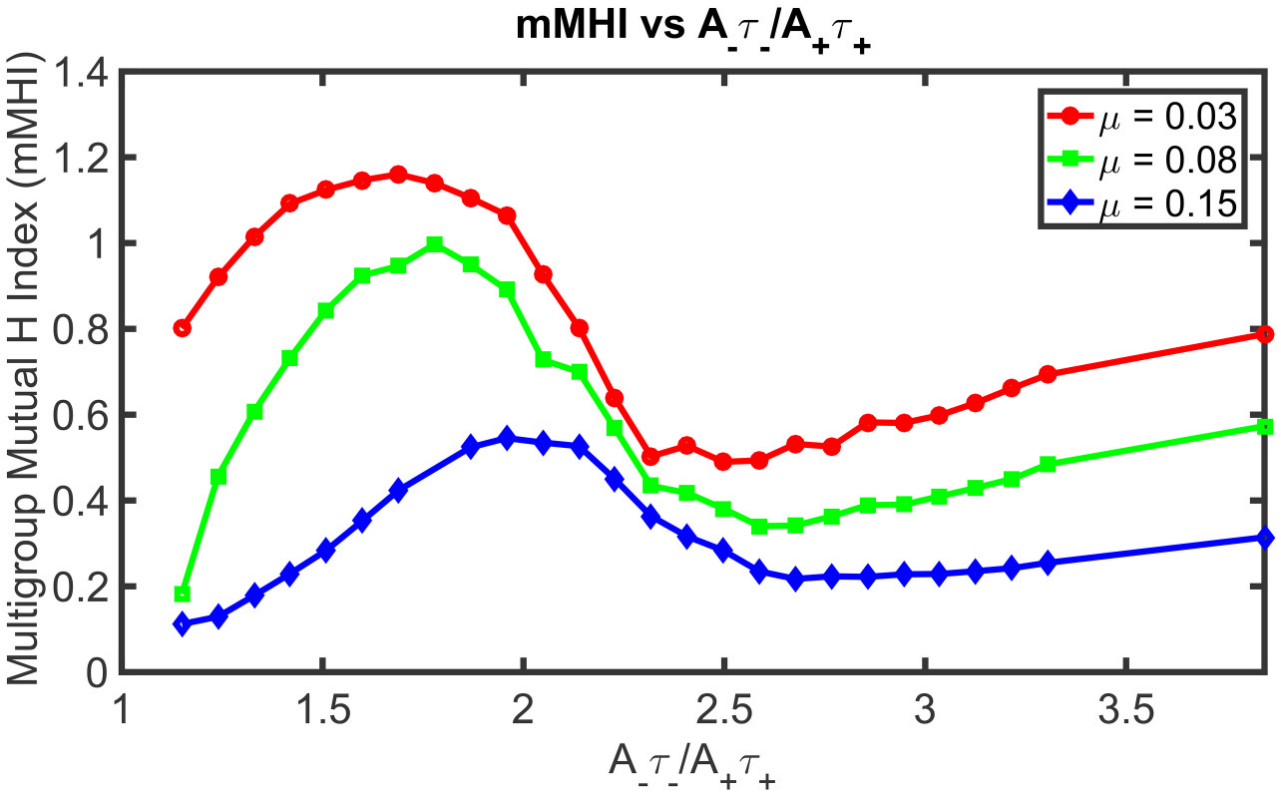
STDP imbalance impacts the dendritic mosaic. Increasing the *A*_−_τ_−_/*A*_+_τ_+_ ratio leads to changes in the mMHI for three different values of the exponent μ appearing in the Gütig STDP model of synaptic competition. Note that we used here the same incremental increases in the value of *A*_−_ as in Figure 2, for the three different values of μ = 0.03, 0.08, 0.15.

Figure 4 shows how STDP imbalance impacts the mMHI for three values of μ = 0.03, 0.08, 0.15, when the neuron is being stimulated by four groups of afferent fibers. Note that the same incremental changes to *A*_−_ used in Figure 2 was adopted. The general shape of the relationship between mMHI and balance ratio is comparable to the one quantified by SDI for two groups of axons and is similar for all values of mu. This dependency is thus robust to change of clustering metric and to increase in the number of inputs, suggesting that this emergent properties could be characterized experimentally (see Section Discussion).

### Mean input frequencies and STDP balance jointly influences the dendritic mosaic

We have shown above that both the degree of synaptic competition and LTP/LTD balance ultimately determines the emergence of the dendritic mosaic. Previously, we have shown that the mean firing frequency of afferent inputs also play an role in this emergence (Iannella et al., 2010).

**Figure 5:**
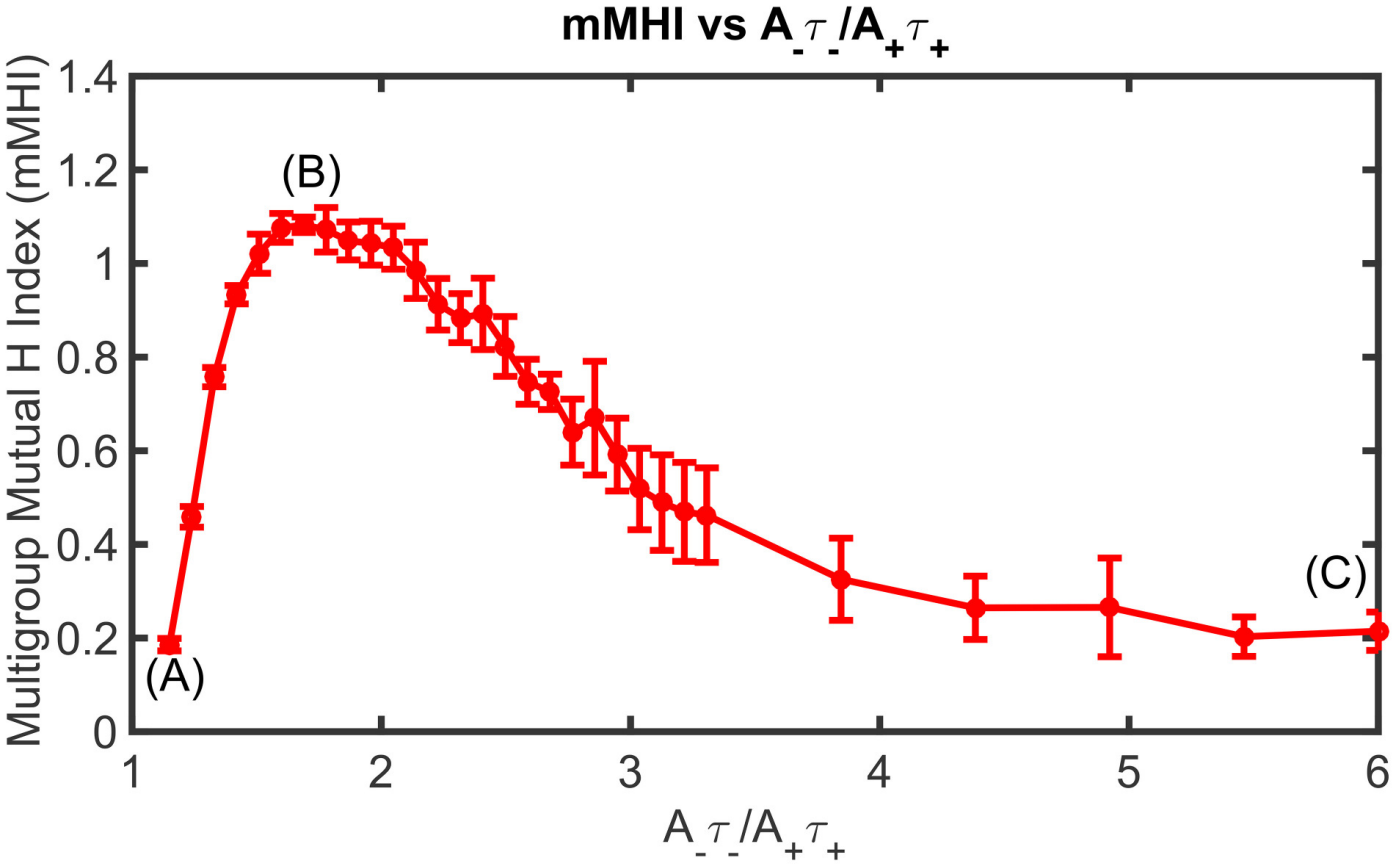
Mean Input Frequency and STDP balance jointly affect the dendritic mosaic. At a given mean input frequency of 10 Hz and μ = 0.03, stepwise increase of *A*_−_τ_−_/*A*_+_τ_+_ produces changes in the mMHI, with a maximum for a ratio of 1.8. Note that (A), (B), and (C) appearing in Figure correspond to balance ratio values of 1.1, 1.8, and 6, respectively.

**Figure 6:**
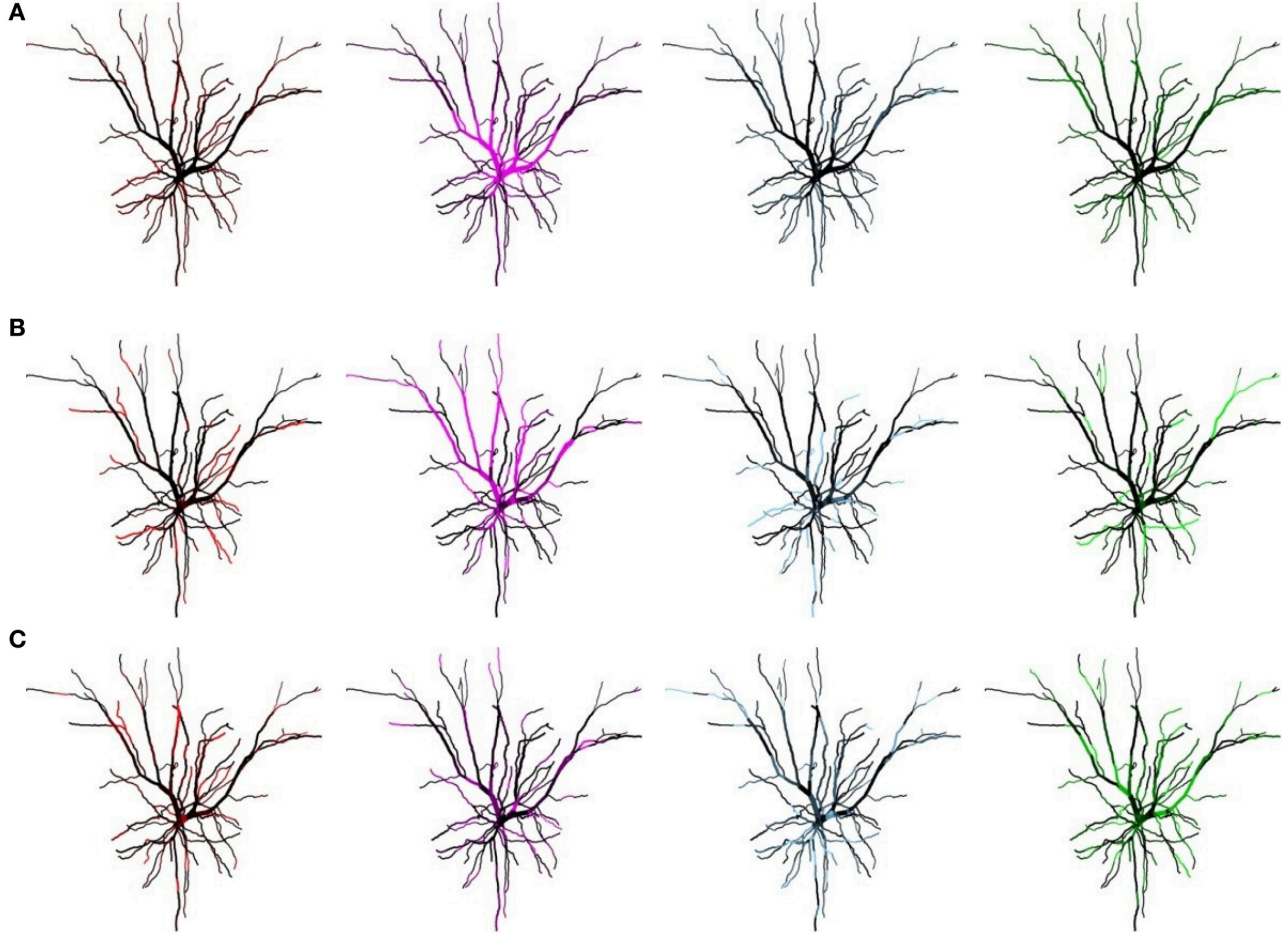
Mean Input Frequency and STDP balance jointly affect the dendritic mosaic. Spatial organization pattern of synaptic efficacies associated with Figure 5 for a balance ratio *A*_−_τ_−_/*A*_+_τ_+_, of 1.1 **(A)**, 1.8, **(B)**, and 6 **(C)**, for each of the four afferent groups. Increasing the balance ratio changes the patterning of synaptic efficacies. Note that while **(C)** corresponds to a low mMHI (Figure 4), the inputs are still clustered, albeit with lower complementarity (i.e., some dendrite segments still show similar synapse strength for different inputs streams after STDP plasticity).

We thus examined the interplay between firing frequency and STDP balance. Figure 5, illustrates how increasing the degree of imbalance affects the mMHI, for a mean input frequency of 10 Hz. Note that in contrast to Figure 4, the mMHI reaches a plateau (≤0.25) at high ratio rather than showing monotonous increase. Equally surprising is the apparent lack of correlation between the low mMHI and the pattern of synaptic efficacy clusters. For a balance ratio of 1.8 (Figure 6, Row B) corresponding to maximal mMHI, we observed intense clustering, with most dendritic compartments being dominated by a single input stream (bright color). Clearly, these inputs have been potentiated through STDP while the inputs from other streams have been depressed.

For lower (A) and higher (C) balance ratio, this pattern is degraded, with a decrease of the contrast between clusters and appearance of overlaps (regions where two or more stream remain strong, thus shown in black). For a balance ratio of 6 (Figure 6C) we even observed subsections of the dendritic tree where efficacy clusters were absent. These dendritic subsections had essentially become regions that do not respond to any group of afferent inputs.

**Figure 7:**
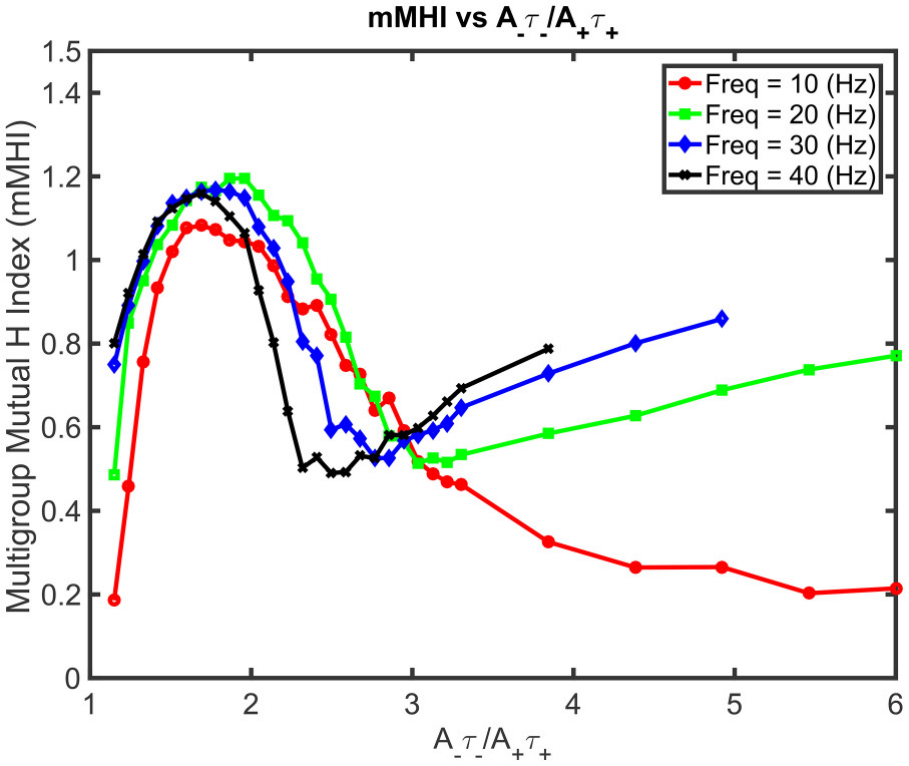
Mean Input Frequency and STDP balance leads to nonlinear effects on mMHI. Increasing both the balance ratio *A*_−_τ_−_/*A*_+_τ_+_ (stepwise as in Figure 2) and the mean input frequency leads to nontrivial changes in the mMHI, with appearance of a local mMHI minimum for higher firing frequencies. Note that the balance ratio at which this minimum is observed seem to depend on the firing frequency.

Increasing both the mean input frequencies and the degree of imbalance induce non-trivial changes for the mMHI (Figure 7). Above 20 Hz, a local mMHI minimum replaces the steady decay seen at lower frequency. The value of the mMHI minimum is approximately independent of frequency but frequency determines the balance ratio at which the minimum occurs. The mMHI maximum remains at 1.8 *A*_−_τ_−_/*A*_+_τ_+_ for all frequencies. Note that already at 40Hz, the upward slope of the mMHI tend toward an asymptotic limit. Indeed no further change were observed at frequencies >40Hz. Thus, the mMHI in the 0 ~ 2.5 *A*_−_τ_−_/*A*_+_τ_+_ range is similar for all input frequencies and below and in the 2.5 ~ 6 range for input frequencies above 30 Hz. This suggests that the conditions required for the optimal tiling of synaptic clustering are robust for a wide range of parameters. The unexpected appearance of a local minimum at ~2.5 may be linked to the degree of local excitability, the number of post-synaptic events generated by incoming inputs and where they occur in dendrites. The number of generated action potentials clearly follows a nonlinear relationship with the mean frequency of the inputs locally targeting the dendrite.

### STDP balance and mean input frequencies jointly influence local spiking

**Figure 8:**
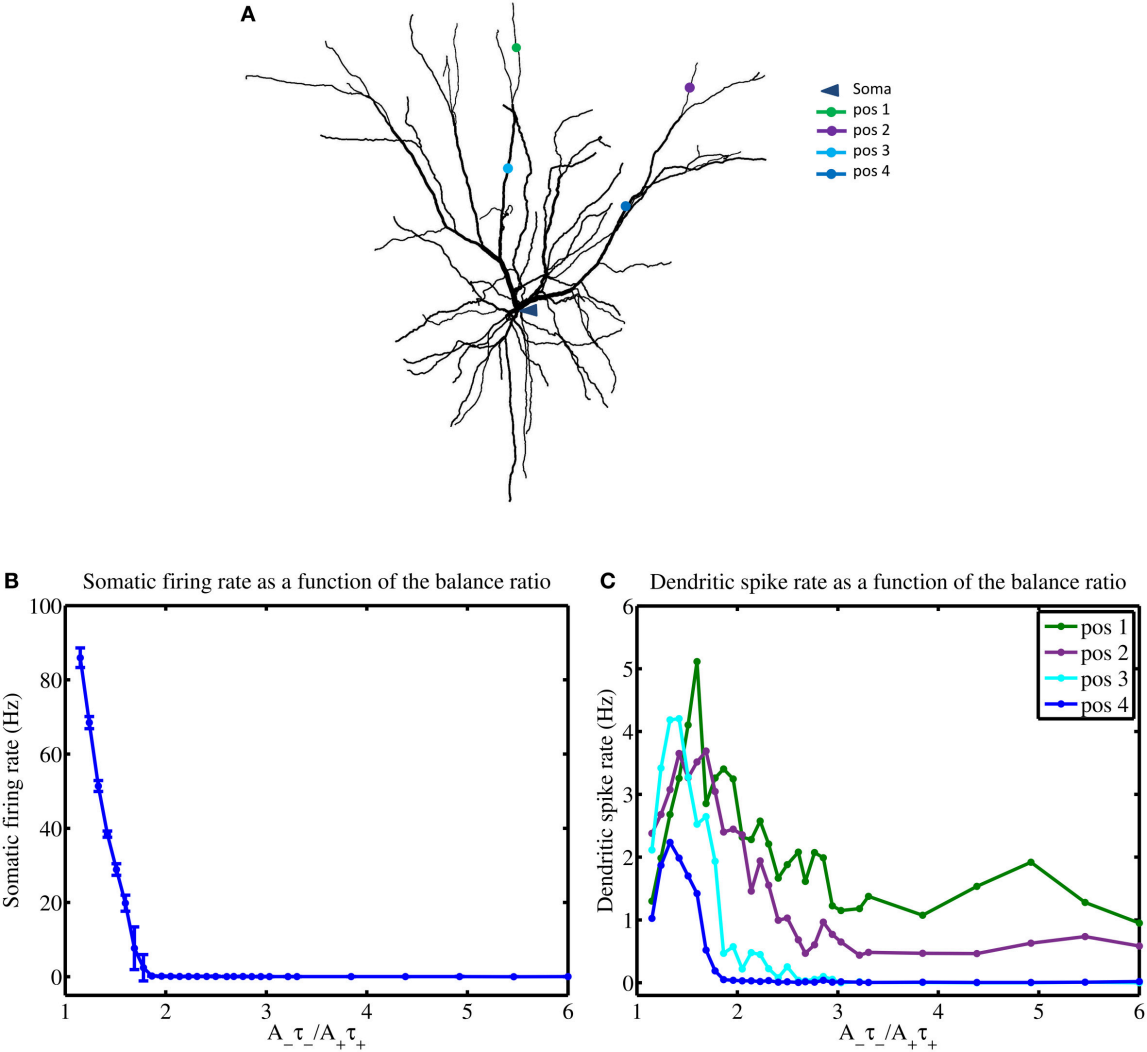
STDP balance affects local spiking. **(A)** Layer 2/3 cell used in simulations indicating the locations of the soma and four distinct dendritic locations denoted by pos 1 through to pos 4, respectively. **(B)** Increasing the *A*_−_τ_−_/*A*_+_τ_+_ balance causes a rapid monotonous decrease of somatic firing rate **(B)** while dendrites show complex responses, measured at 4 different locations (pos 1–4) **(C)**, using a mean input stimulus frequency of 10 Hz. All four dendritic locations show a spiking maxima for the balance ratio resulting in maximal mMHI (1.8) but only approximately and with marked differences. Notably, for larger values of the balance ratio, the dendritic firing rates reduce to zero for positions 3 and 4, while the other two locations show sustained nonzero rate.

In the previous section, we saw how the mean input frequencies and the degree of STDP balance affects the emergence of the dendritic mosaic. Here, we analyze the corresponding alterations to local neuronal response at the soma and several representative dendritic locations, as shown in Figures 8A,B. Firing rate at the soma (when subjected to inputs from all four afferent groups) dramatically decreases from 86 Hz to 0.2 Hz as the degree of imbalance (balance ratio) increases from 1.05 through to 1.8 (Figure 8A).

For larger degrees of imbalance (from 1.8 to 6) the neuronal firing rate remains relatively constant around 0.03 Hz. In contrast, the firing rate for the dendritic locations display maximal spikes rates at different values of the balance ratio (Figure 8B) and different level of activity, even when LTD is favored (high STDP imbalance ratio).

### Influence of morphology

In the realistic model presented above, we have measured how STDP balance and mean input frequency influence the emergence of efficacy clusters. Since clustering emerge from local interactions of synaptic inputs, we wondered to what extend dendritic morphology plays in the appearance and stability of the mosaic. Historically, Rall (1964) was the first to show how neural morphology can influence the firing properties of neurons, using simple compartmentalized ball-and-stick models (Rall, 1964). To directly investigate whether the morphology of the dendrites impacts the emergence of the dendritic mosaic, we chose to compare a realistic (reconstructed from tracing) neuron morphology to an extremely simplified simple cable equivalent. This extreme approach allowed us to preserve the active properties of the original cell with only simple transformations while reducing the morphology to an unbranched dendrite. We thus generated the simplified models using two published reduction methods that map complex dendritic morphologies to an unbranched cable structure while maintaining axial resistance and without altering the active properties used in the original model. An alternative approach could be to systematically change the lengths and diameters of dendritic branches, or to gradually merge branches together until significant mosaic alterations are detected. This approach has the double handicap of being both computationally intensive and not amenable to experimental testing. Instead, in a future study, we intend to compare mosaic formation in realistic but different morphologies such as CA1 pyramidal cell, Purkinje cell and spinal cord motoneurons and compare simulation data to physiological data.

**Figure 9:**
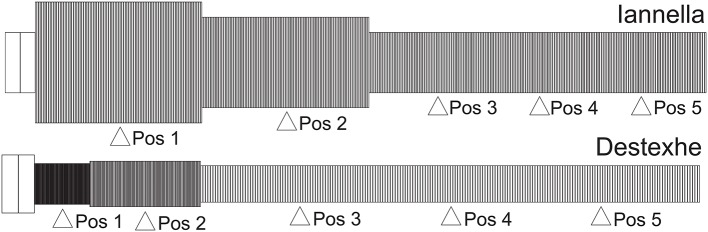
Unbranched cable morphology. Application of the reduction schemes proposed by us (Iannella et al., 2004) and by Destexhe et al. (1998) to transform the original dendritic tree into an unbranched cable composed of three sections, each with different radii. Pos 1, 2, and 4 are at the middle of each section while Pos 3 and 5 are at 0.2 and 0.8 of the most distal section.

The first of these methods relies upon a very simple construction yielding a simplified equivalent cable morphology of the original dendritic tree, consisting of only three identified regions but conserving the electrical properties, length and total surface area of the original cell (Iannella et al., 2004), as shown in Figure 9. The second method is based upon combining branches into an equivalent cylinder where the axial resistance of the original branches are not altered (Destexhe et al., 1998). This requires summing the cross sectional area of each contributing branch and results in an equivalent cylinder with a radius given by the square root of sum of all contributing radii squared, $r=\sqrt{\underset{i}{\sum }r_{i}^{2}}$. The length of the cable is taken to be the average length of contributing branches weighted by their respective radii *r*_*i*_, in order to take different contributing branch lengths into account, $l=\underset{i}{\sum }l_{i}r_{i}/\underset{i}{\sum }r_{i}$. The resulting total surface area of the simplified model differs from the original, requiring correction of all conductances and the membrane capacitance by a multiplicative factor to maintain input resistance (see Destexhe et al., 1998 for more details). The Destexhe simplified morphology is presented in Figure 9.

**Figure 10:**
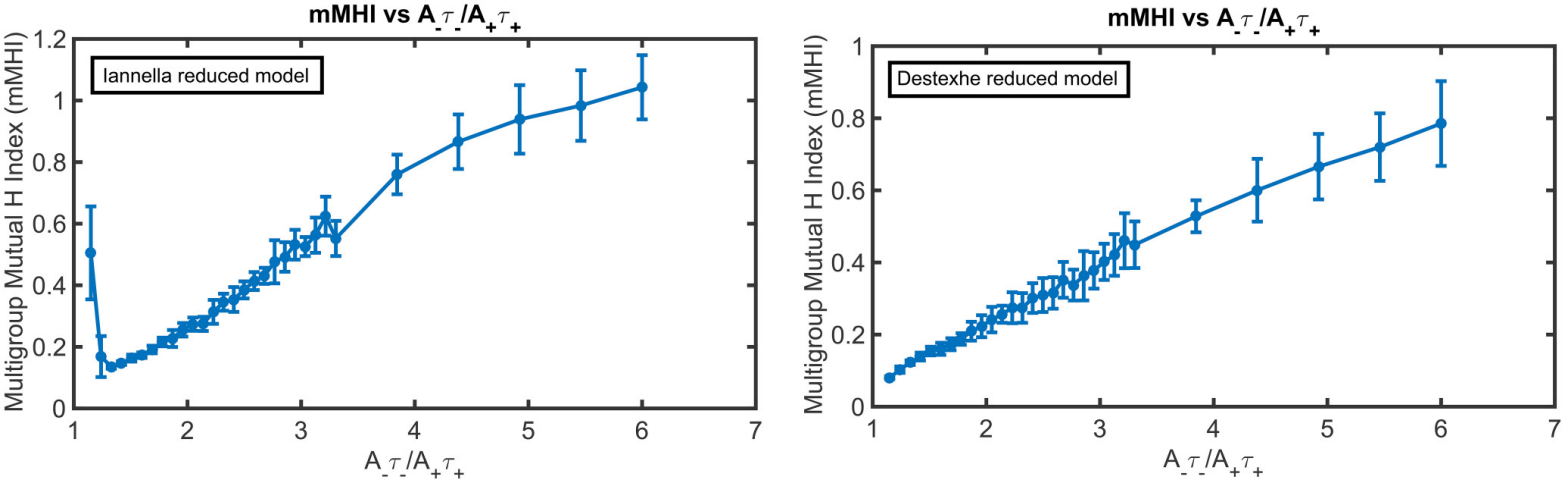
The mMHI in reduced models. mMHI is computed for the balance ratio *A*_−_τ_−_/*A*_+_τ_+_ ranging from 1.15 to 6.0, using a mean input stimulus frequency of 40 Hz. Note that mMHI was more densely sampled for balance ratio between 1.15 to 3.3, as in Figure 2.

Figure 10 presents the mMHI index as a function of STDP imbalance for both of these simplified models. Altering the morphology while keeping the same active properties alters the mMHI (Compare to Figure 2). Notably, for the first method, there is an initial sharp drop followed by a slower monotonic rise in the mMHI index while for the second method, mMHI is a monotonic function of balance. Both mMHI profiles are less complex than profile obtained for the complex model (Figure 2), showing that dendritic morphology plays an important role in the emergence of the dendritc mosaic and its constituent synaptic efficacy clusters.

Increasing the degree of imbalance (by increasing the balance ratio) leads to different quantitative effects on the resulting spatial patterning of synaptic efficacy for each simplified model. For the Iannella reduced model one clearly sees that for an initial balance ratio value of 1.15 there is a preference to form large spatial clusters that occupy distinct portions of the unbranched dendrite, thus indicating that for this balance ratio, STDP implements an underlying spatial winner-take-all process that allow large spatial clusters to emerge. The formation of large spatial clusters, however, is a transient occurrence since a small increase in the balance ratio from 1.15 to 1.33 leads to the emergence of smaller clusters spread throughout the dendrite that can overlap in space with other clusters contributed by other groups.

This is in stark contrast to the formation of large distinct clusters. The appearance of large spatial clusters usually indicates synergy between synapses however the transformation from large to small clusters signifies a sudden loss of spatial extent of co-operativity between synapses. Increasing the balance ratio further leads to small synaptic efficacy clusters that are sparsely distributed but can overlap with other efficacy clusters contributed by other afferent groups. In comparison, the Destexhe simplified model results in only small clusters that are spread out throughout the entire extent of the unbranched dendrite, but as the balance ratio is increased, this gives rise to small efficacy clusters that can spatially overlap with other clusters (contributed by other afferent groups) but are also freely distributed along the extent of the dendrite.

**Figure 11:**
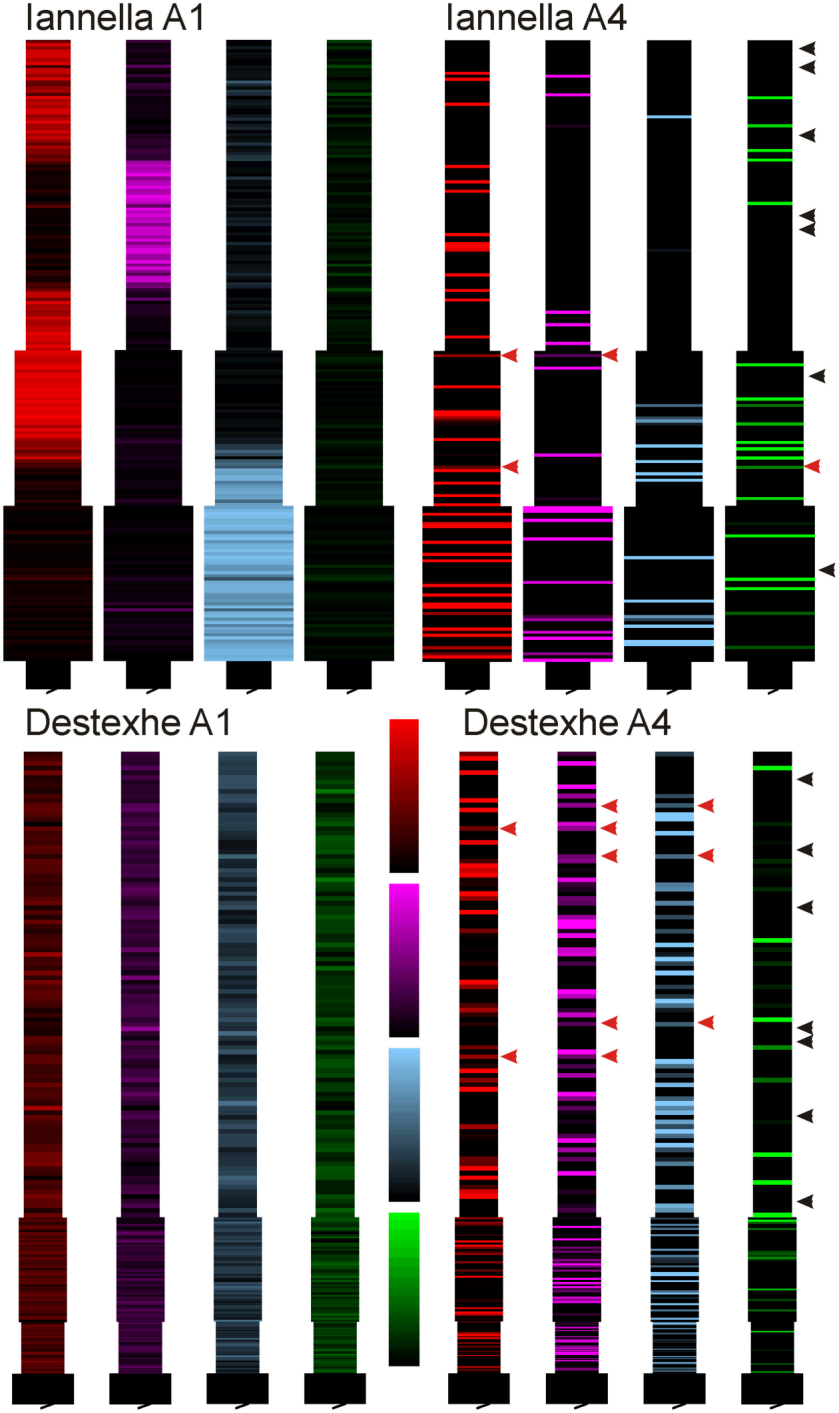
Synaptic efficacy clusters in simplified model. Spatial organization pattern of synaptic efficacies associated with Figure 10 for balance ratios *A*_−_τ_−_/*A*_+_τ_+_, of 1.15 (A1) and 6.0 (A4), for each of the four afferent groups. Increasing the balance ratio has qualitatively different effect on the two simplified models (compare A1), but high ratio yields clear local clustering for both models (A4). Note that at high ratio, some regions are essentially devoid of dominant input (black arrowheads) while some regions show mixed dominance by two or more inputs (red arrowheads).

Inspecting the spatial organization of clusters that have emerged in both simplified models reveals some interesting common traits. For both simplified models, increasing the degree of imbalance leads to the formation of small localized synaptic efficacy clusters that are sparsely distributed in dendritic space. Notably, when balance ratio is large, there are spatial regions in the dendrite that are devoid of any input (see Figure 11 these are indicated by black arrowheads), while in other regions there can be two or more localized clusters that overlap, potentially mutually augmenting their inputs (see Figure 11 indicated by red arrows).

**Figure 12:**
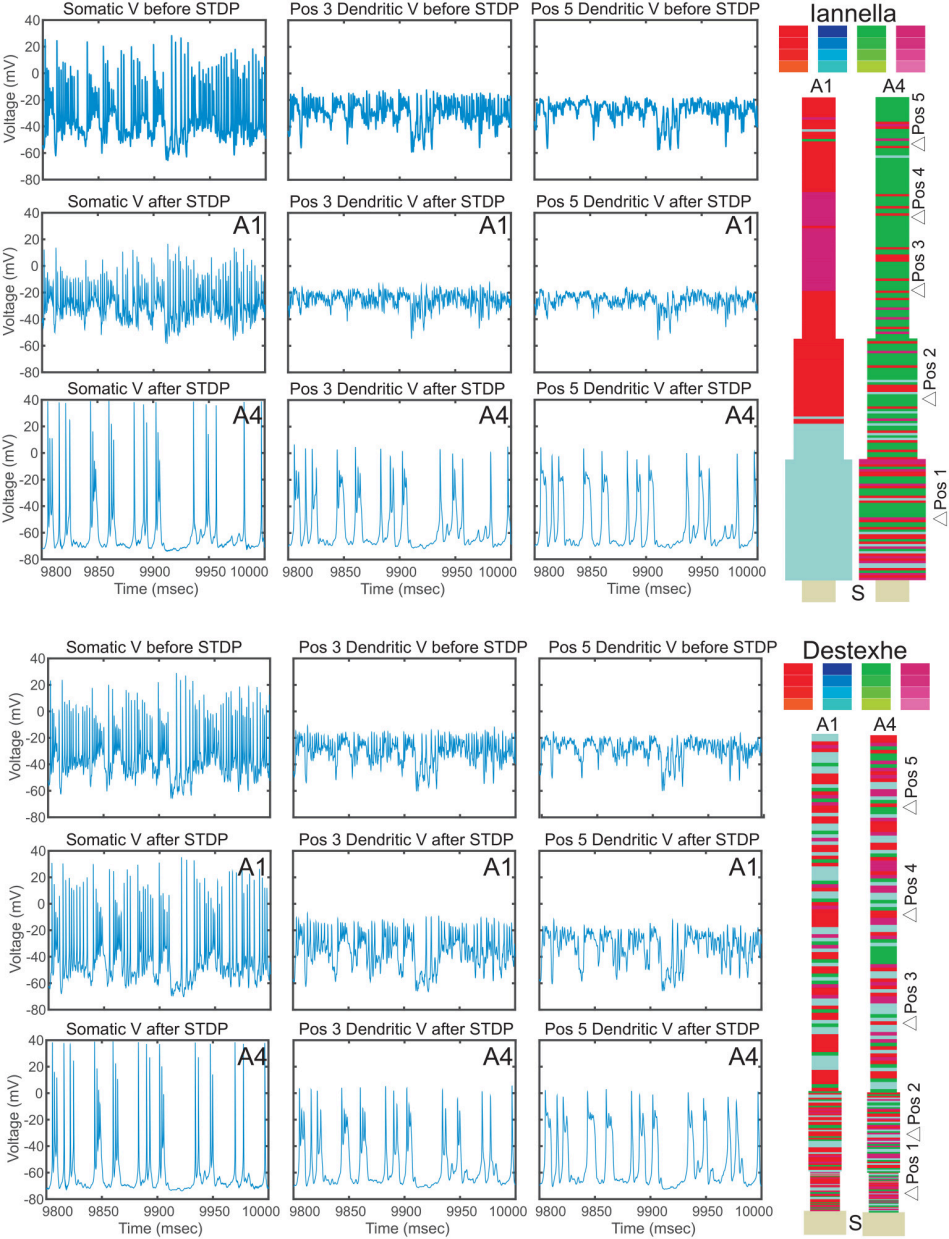
Electrical activity before and after STDP. Traces of the membrane potential recorded from the soma and dendritic locations Pos 3 and Pos 5 before and after the of STDP, for two different values of STDP balance denoted by A1 = 1.15 and A4 = 6. After STDP, the membrane potential tends to the resting potential value, with spikes or spike bursts occurring irregularly. Note that the two simplified models converge to remarkably similar pattern of activity, despite the markedly different synaptic mosaic (see Supplementary Materials for other locations).

Since there is a drastic change of synaptic efficacy, we wondered about the concurrent alterations the neuron's input-output relationship in both the space and time domains. Samples of the membrane potential recorded from three different locations, the soma and two different dendritic locations respectively labeled as “S,” “Pos 3,” and “Pos 5” as indicated in Figure 12 before and after STDP learning for both simplified models. Figure 12 illustrates these membrane potential traces for each model at two different values of the balance ratio $\frac{A_{-}\tau_{-}}{A_{+}\tau_{+}}\;=\;1.15$ and $\frac{A_{-}\tau_{-}}{A_{+}\tau_{+}}=6.0$ denoted by *A*1 and *A*4, respectively. Here, when a balance ratio of 1.15 is used, one can observe small qualitative changes to the membrane potential at the above described locations after STDP. Conversely, a large balance ratio value leads to marked qualitative alterations to these membrane potential traces after STDP (*A*4). Specifically, the membrane potential tends to the resting state, with the occasional occurrence of spikes or burst of spikes, at the three specified locations.

## Discussion

In this study, we investigated how synaptic competition and STDP jointly determines the formation and stability of clustered synaptic efficacy engrams in a realistic biophysical model. Similar to our previous study (Iannella and Tanaka, 2006), when the model received inputs from two groups of correlated afferent fibers (with no inter-group correlation), STDP learning results in the formation of interdigitated regions of synaptic efficacy clusters, forming a spatially complementary pattern of synaptic strength for each respective group of afferents. With four groups of afferent fibers (again with intra-group correlation), we still observe the emergence of the dendritic mosaic as a result of STDP learning. Here we examined the relative contribution and influence of mean input frequency, the degree of synaptic competition, and the balance between potentiation and depression, in this phenomenon.

Considering the non-linear dynamic of the system under study (a spatially extended model with full complement of ion channels) and the stochasticity introduced through the random arrival times of the afferent inputs, analysis of parameters contributions to spatial pattern formation is not possible analytically. Here we addressed the issue by an exhaustive exploration of the parameter space through simulation, both in a complex model and in a model with simplified morphology but similar electrophysiological properties.

One of the key features used of the model is that it detects local dendritic spikes to convey post-synaptic timing information locally, as opposed to the global nature of the back propagating action potential (BPAP). Whether BPAPs can fulfill the role of telling every synapse in the dendrite when the neuron fired an action potential has come into question. Experiments have shown that the BPAP does not fully invade the dendrite due to voltage attenuation (Larkum et al., 2001; Stuart and Häusser, 2001) and that synaptic activity either reduce or block BPAP invasion into the dendrite completely (Paré et al., 1998; Mickus et al., 1999; Larkum et al., 2001). In addition, changes in synaptic efficacy can occur without the need of a BPAP (Schiller et al., 2000; Golding et al., 2002; Holthoff et al., 2004) and constant synaptic bombardment across the dendrite can cause spike generation in dendrites, which may also limit BPAP propagation (Paré et al., 1998; Larkum et al., 2001). Therefore, it seems unlikely that the BPAP could provide every synapse located within the dendritic tree with the necessary timing information of when postsynaptic firing occurred. Post-synaptic timing information carried by locally triggered dendritic spikes may provide a more robust signal. From a theoretical standpoint, the use of dendritic spikes may play an important computational role in permitting the neuron to develop functional compartments, allowing the neuron to perform complex computations or increase its memory storage capacity (Poirazi et al., 2001, 2003b; Polsky et al., 2004).

A defining feature of our model is the emergence under STDP of synaptic clusters forming a dendritic mosaic (tessellation). This was observed over a restricted region of the multidimensional parameter space defined by (1) the degree of synaptic competition, (2) mean input frequency, and (3) the amount of synaptic balance. In addition, we found that when STDP learning is dominated by depression, learning still gives rise to synaptic clusters despite the imbalance introduced between depression and potentiation components of the temporal learning window. Note however that the spatial organization of these clusters fail to form a continuous tiling pattern in some regions of the dendrites. This result in degraded dendritic mosaic, with areas essentially devoid of synaptic inputs. Functionally, this may correspond to regions of silent synapses or the synaptic cold-spot previously described experimentally (Zador et al., 1992). Finally, comparison with simple model derived from the realistic one shows degraded ability to form a mosaic, confirming the role of local non-linearities. Specifically, our model layer 2/3 pyramidal cell was compared to two equivalent cable models (whose reduction has been detailed elsewhere, Destexhe et al., 1998; Iannella et al., 2004) that conserve input resistance of the original neuron. Both full and reduced models used identical sets of ion channels. After STDP learning, analysis of the mutual information index and of the corresponding synaptic clusters shows an altered mosaic formation, for a wide range of STDP balance. These differences arise since altering the morphology of the dendrites ultimately changes how synaptic inputs interact with each other and with local channels in dendrites. This leads to non-linear inputs summation and local alteration of input resistance, thus altering the local conditions of STDP.

Our study provide new insight on the interplay between synapse location, active dendritic properties, morphology and synaptic plasticity in shaping the strengths and spatial arrangements of synapses. One strong prediction arising from this study is that “inputs clustering” may be the favored and natural outcome of synaptic plasticity when neurons receive different streams of correlated inputs, as often seen in sensory and associative brain areas. Indeed, in the visual and auditory systems (and probably other sensory systems), clustering functionally related inputs on different dendritic domains seem to play a role in tuning the neuron for contrasting stimuli in time and/or spac (McBride et al., 2008; Jia et al., 2010; Kleindienst et al., 2011; Podgorski et al., 2012). Our results indicate that maximum clustering -and thus the ability to discriminate stimuli- emerge and is only produced and maintained for a narrow band of input frequency. This implies that there is conflict between building the ability to discriminate between stimuli (through STDP) and the ability to encode stimuli intensity as spike rate. Indeed, our results predict that a neuron exhibiting mosaic clustering should exhibit approximately constant EPSP frequency when stimulus intensity varies widely. This limitation only exists however if STDP is maintained throughout life. If the mosaic is formed during a developmental critical period, before “crystallization” in the adult then the same neuron would exhibit both synaptic mosaic (input discrimination) and ability to integrate rat-coded signals. We predict that genetic or pharmacological manipulation of plasticity balance in the young, typically by changing the level of GABA inhibition (Hensch et al., 1998; Morales et al., 2002; Takesian and Hensch, 2013), homeostatic mechanisms (Turrigiano and Nelson, 2004), the activity of NMDA receptors (Medina et al., 1999; Quinlan et al., 1999; Krapivinsky et al., 2003; Bender et al., 2006; Nevian and Sakmann, 2006), should permanently impair formation of cluster, with the correlate that this would diminish the ability to detect contrast stimuli in the adult.

Comparing the profiles of somatic and dendritic firing rates (Figure 8) with the mMHI (Figure 8), it appears that the non-monotonic variations of mMHI with increased imbalance may result from the interplay between membrane excitability and the exposure to STDP. This results in a large reduction of the neuron's (somatic) firing rate after STDP learning, from 86 Hz to 0.2 Hz to 0.03 Hz. The reason is that STDP net effect is to reduce synaptic weights. Interestingly, an emergent feature in this context is the presence of dendritic regions that seem to resist being silenced (enduring the suppressive nature of STDP due to increasing balance ratios), and responding with higher rates of spike generation than those observed at the soma. This suggests that these particular regions of the dendrite essentially behave as functional subunits, providing the neuron with additional levels of processing (similar to what one expects from a neural network) before global integration and spike firing takes place in the soma (albeit at a diminished rate). Put simply, one observes the emergence (via STDP) of neuron that functionally behaves more as a two-layer (or more) distributed network rather than as a globally weighted summation device. This view is in agreement with the view that dendritic branches can potentially behave as independent functional units (computational subunits) and secondly, that they may promote functional compartmentalization of inputs in dendrites (Poirazi et al., 2003b; Losonczy and Magee, 2006; Harvey and Svoboda, 2007; De Roo et al., 2008; Larkum et al., 2009; Govindarajan et al., 2011; Kleindienst et al., 2011; Legenstein and Maass, 2011; Makino and Malinow, 2011; Harnett et al., 2012; Major et al., 2013; Sajikumar et al., 2014). It is important to note, however, that dendritic branches behaving as computational subunits tend to be dynamic in nature where the spatial and temporal patterning of inputs and the nonlinear nature of the dendritic membrane drives the functional properties of dendritic integration.

Furthermore, the firing profiles at different dendritic locations (Figure 8) are the result of generating dendritic spikes at different rates and depend on the nonlinear nature of the dendritic channels and local branching morphology. The clustering results in tuning various portions of the dendritic tree in an unsupervised manner. This self-organization process causes specific portion of the dendritic tree to become maximally responsive to specific inputs. This is akin to the branch specific plasticity recently described by Losonczy et al. (2008), Kleindienst et al. (2011), Legenstein and Maass (2011), Makino and Malinow (2011), and Sajikumar et al. (2014). These results thus point to previously not fully unrecognized properties of dendrites, allowing different sections of the dendritic tree to be “selectively tuned” through synaptic plasticity processes that take into account the nonlinear nature of dendritic voltage depolarization and the statistical structure of the inputs. Such selective tuning may allow the dendrites to process inputs independently and to compartmentally store input features in a robust manner. This added complexity yield a more dynamic and nontrivial computational model of neuronal processing and input/output responses.

### Relation to other models

There is renewed interest in investigating how synaptic plasticity applied to passive or active dendritic trees shapes the storage of input features via the formation of dendritic compartmentalization (Rabinowitch and Segev, 2006a,b). For example, Tamosiunaite et al. (2007) have reported a type of winner-take-all competition between dendritic branches. In contrast to our study and that of Legenstein and Maass (2011), they only considered the case where individual input groups were initially spatially segregated and would target single branches (Tamosiunaite et al., 2007). More realistically, Legenstein and Maass (2011) have directly implemented neuronal mechanisms where both functional compartmentalization of input features takes place and dendritic branches compete and behave as individual computational subunits. These authors have shown that when explicitly incorporating two levels of competition, one between dendritic branches and the other at the winning branch between locally situated synapses, correlated synaptic activity is strengthened while the efficacy of other synapses decays with time. This permits the features of single input pattern to be stored locally even in one single branch. This type of self-organization thus allows the storage of multiple input features by a single neuron in different non-overlapping regions of the dendrite.

### Mechanisms underlying inhomogeneous spatial patterns of clusters

The functional benefits of spatially organized synaptic inputs are well documented (Rall, 1964; Mel, 1992b). The precise biophysical mechanisms leading to this organization, allowing memory engram to be represented in “synaptic clusters” are thus of prime interest, both from biological and theoretical perspectives. Previous studies of neural networks self-organization into functional maps have shown that non-linear interaction of short-range excitation and longer-range inhibition are fundamental in the emergence of any type of clustered organization (Haken, 1977; Kohonen, 1982; Haken, 1983). This interaction is important since it sets up a winner-take-all mechanism which, under the appropriate conditions, ultimately leads to the development of the functional clusters thus forming the map. The interaction function is spatial in nature and usually balanced in the sense that positive and negative areas of the function are (nearly) equal and thus balance each other out. This type of interaction is also envisioned to occur in dendritic trees.

### Limitations of the study

Our study describes the conditions under which synaptic efficacy clusters may emerge to form a dendritic mosaic. We found that it is jointly determined by multiple factors, including mean input frequency, the degree of synaptic competition, synaptic balance and dendrite morphology. The region in this multidimensional parameter space where both synaptic efficacy clusters and the dendritic mosaic emerge as a result of STDP learning correspond to a physiological range (frequency) or range used by others. Future work may consider investigating the role of synaptic balance when both excitatory and inhibitory synapses undergo plastic change, to elucidate how balance and plastic inhibitory synapses jointly impact the spatial organization of both excitatory and inhibitory synaptic efficacies.

A novel extension may be to investigate other forms of morphological influences. Recent studies have pointed out additional influence of neuronal morphology on synaptic plasticity and on formation of cortical circuits. For example, important differences in the shape and distribution of dendritic spines along neuronal dendrites between pyramidal cells from different cortical areas, layers, and species have been observed (Murayama et al., 1997; Elston, 2003; Bianchi et al., 2013; Elston and Manger, 2014). In primates, for example, both the numbers, density, and distribution of dendritic spines differ for pyramidal neurons in different cortical areas, while in the mouse the spine density seems to be constant (Murayama et al., 1997; Elston, 2003; Ballesteros-Yáñez et al., 2006; Benavides-Piccione et al., 2006; Bianchi et al., 2013; Elston and Manger, 2014). Another aspect are dendritic spines, as these are the loci where the formation and refinement of cortical circuitry through processes of synaptic plasticity and (consequently) synaptic transmission takes place. In basal dendrites, it has been reported that the size of the spine head is proportional to the number of post-synaptic receptors and pre-synaptic docked vesicle, while the length of the spine neck seems to be associated with the calcium compartmentalization (Ballesteros-Yáñez et al., 2006). Taken together, all these point to the need to re-examine the role played by neuronal morphology in brain development, especially the impact of developmental morphological changes, synaptic plasticity, and synaptogenesis has on the formation of cortical circuits including the patterning of convergent afferent inputs to neurons. Although such a study would be highly valuable, care needs to be taken to incorporate biophysically meaningful processes that correctly capture the biochemical processes of activity-dependent synaptic plasticity, neuronal growth, and spine creation and elimination. The results of such a study will be presented in a future publication and is thus beyond the scope of this paper.

Another extension would be to base the STDP plasticity on an explicit biophysical model of calcium dynamics and biochemical signaling cascades involved in learning and memory. One candidate is the plasticity rule first introduced by Graupner and Brunel (2007). Using such a rule would allow to correlate the emergence of synaptic efficacy clusters with the underlying states of the biochemical signaling cascade, generating experimentally testable predictions. Other potential improvements are an explicit modeling of dendritic spines and of the underlying reaction-diffusion processes, along with Graupner's calcium-based plasticity rule (Graupner and Brunel, 2007). We expect that the spatially restricted calcium signals may allow the emergence of synaptic mosaic for more streams of correlated inputs, beyond the two and four groups tested in the current model.

## Conclusions

This current study illustrates how the level of synaptic balance, admitted by STDP, impacts the formation of synaptic efficacy clusters in dendrites. We believe that the current study provides useful insights for the interplay between synapse location, synaptic plasticity, and the active properties of the membrane not only shapes the strengths and spatial arrangements of synapses but highlights the emergence of a functional compartmentalization from STDP. Furthermore, we also illustrated that cellular morphology can play a significant role in the emergence of efficacy clusters. In particular our results hint that dendritic branches, under the right conditions, can act as (near) independent functional units, in agreement with other authors (Losonczy et al., 2008; Kleindienst et al., 2011; Legenstein and Maass, 2011; Makino and Malinow, 2011). This permits a novel subdivision of dendritic space and paves the way for the formation of selectively responsive regions of the dendrite and further suggests that the distributed storage of information is the natural mode of information storage in neural circuits. Finally, we are considering further extensions to the current research, such as the inclusion of more detailed biochemistry, dendritic spines, and reaction diffusion processes. These extensions would permit a deeper understanding, at the subcellular level, into how the interplay between synapse location, calcium based biochemistry, and synaptic plasticity in neuronal dendrites shapes dendritic information storage within neural circuits.

## Author contributions

Conceived the simulations, analyzed the data, and wrote the manuscript: TL and NI. Performed the simulations: NI.

### Conflict of interest statement

The authors declare that the research was conducted in the absence of any commercial or financial relationships that could be construed as a potential conflict of interest.
