# Supplementary material for: Modulating STDP Balance Impacts the Dendritic Mosaic
Source: Front Comput Neurosci. 2017 Jun 9;11:42. doi: 10.3389/fncom.2017.00042 (PMC5465290; doi:10.3389/fncom.2017.00042)
Supplement: Supplementary file 1 [file Presentation1.pdf]

# *Supplementary Material*: Modulating STDP balance impacts the dendritic mosaic

**Nicolangelo Iannella** [1,2*], **Thomas Launey** [3]
*Correspondence:
[1] School of Mathematical Sciences, The University of Nottingham, University Park, Nottingham NG7 2RD, United Kingdom
[2]Computational and Theoretical Neuroscience Laboratory, Institute for Telecommunications Research, University of South Australia, Mawson Lakes, South Australia, Australia
[3]Lab. Synaptic Molecules of Memory Persistence, RIKEN, Brain Science Institute, 2-1 Hirosawa Wako-shi, Saitama 351-0198, Japan Nicolangelo Iannella
Email: nicolangelo.iannella@gmail.com

## 1 SUPPLEMENTARY DATA: LAYER 2/3 PYRAMIDAL CELL MODEL DETAILS

We present the set of ionic currents used in our layer 2/3 pyramidal cell model. The descriptions of the ionic currents used in the simulations were the same or similar to those used in previous modeling studies (**Iannella et al.**, 2010; **Iannella and Tanaka**, 2006; **Iannella et al.**, 2004; **Mainen et al.**, 1995; **Rhodes and Gray**, 1994; **Rhodes and Llinás**, 2001; **Traub et al.**, 2003) and are given below. Note that the values of maximal conductances for each respective ion channel are listed below.

The values of maximal conductances for each respective ion channel are listed in Table 1. at the end of this document.

*Leak current* $I_{\mathrm{leak}}$

$$I_{\mathrm{leak}} = g_{\mathrm{l}}(E_{\mathrm{leak}} - V)$$

*Sodium current* $I_{\mathrm{Na}}$

$$\begin{aligned}
I_{\mathrm{Na}} &= \overline{g}_{\mathrm{Na}} m^3 h (E_{\mathrm{Na}} - V) \\
\frac{\partial m}{\partial t} &= \alpha_{\mathrm{m}}(V)(1-m) - \beta_{\mathrm{m}}(V)m \\
\alpha_{\mathrm{m}}(V) &= 0.182(V+35)/(1-\exp(-(V+35)/9)) \\
\beta_{\mathrm{m}}(V) &= -0.124(V+35)/(1-\exp((V+35)/9)) \\
\frac{\partial h}{\partial t} &= \frac{h_\infty(V) - h}{\tau_{\mathrm{h}}(V)} \\
\alpha_{\mathrm{h}}(V) &= 0.024(V+50)/(1-\exp(-(V+50)/5)) \\
\beta_{\mathrm{h}}(V) &= -0.0091(V+75)/(1-\exp((V+75)/5)) \\
h_\infty(V) &= 1/(1+\exp((V+65)/6.2)) \\
\tau_{\mathrm{h}}(V) &= 1/(\alpha_{\mathrm{h}}(V) + \beta_{\mathrm{h}}(V))
\end{aligned}$$

*Potassium current* $I_{\mathrm{K}}$

$$
\begin{aligned}
I_{\mathrm{K}} &= \bar{g}_{\mathrm{K}} n (E_{\mathrm{Kdr}} - V) \\
\frac{\partial n}{\partial t} &= \alpha_{\mathrm{n}}(V)(1-n) - \beta_{\mathrm{n}}(V) n \\
\alpha_{\mathrm{n}}(V) &= 0.02(V-20)/(1-\exp(-(V-20)/9)) \\
\beta_{\mathrm{n}}(V) &= -0.002(V-20)/(1-\exp((V-20)/9))
\end{aligned}
$$

*Transient potassium A-current* $I_{\mathrm{K(A)}}$

$$
\begin{aligned}
I_{\mathrm{K(A)}} &= \bar{g}_{\mathrm{K(A)}} m^4 h (E_{\mathrm{K(A)}} - V) \\
\frac{\partial m}{\partial t} &= \frac{m_\infty(V) - m}{\tau_{m_{\mathrm{K(A)}}}(V)} \\
\frac{\partial h}{\partial t} &= \frac{h_\infty(V) - h}{\tau_{h_{\mathrm{K(A)}}}(V)} \\
m_\infty(V) &= 1/(1+\exp(-(V+60)/8.5)) \\
h_\infty(V) &= 1/(1+\exp((V+78)/6)) \\
\tau_{m_{\mathrm{K(A)}}}(V) &= 0.185 + 0.5/[\exp((V+35.8)/19.7) + \exp(-(V+79.7)/12.7)] \\
\tau_{h_{\mathrm{K(A)}}}(V) &= 0.5/[\exp((V+46)/5) + \exp(-(V+238)/37.5)] \qquad \text{for } V \le -63 \\
&= 9.5 \qquad \text{for } V > -63
\end{aligned}
$$

*Potassium H-current* $I_{\mathrm{K(H)}}$

$$
\begin{aligned}
I_{\mathrm{K(H)}} &= \bar{g}_{\mathrm{K(H)}} m (E_{\mathrm{K(H)}} - V) \\
\frac{\partial m}{\partial t} &= \frac{m_\infty(V) - m}{\tau_{m_{\mathrm{K(H)}}}(V)} \\
m_\infty(V) &= 1/(1+\exp((V+75)/5.5)) \\
\tau_{m_{\mathrm{K(H)}}}(V) &= 1/[\exp(-0.086V - 14.6) + \exp(-1.87 + 0.07V)],
\end{aligned}
$$

*High-Voltage-Activated (HVA) L-type calcium current* $I_{\mathrm{Ca(HVA)}}$

$$
\begin{aligned}
I_{\mathrm{Ca(HVA)}} &= \bar{g}_{\mathrm{Ca(HVA)}} m^2 h \left([\mathrm{Ca}]_i\right) \left(E_{\mathrm{Ca(HVA)}} - V\right) \\
E_{\mathrm{Ca(HVA)}} &= \frac{R\mathcal{T}}{2\mathcal{F}} ln \left(\frac{[\mathrm{Ca}]_o}{[\mathrm{Ca}]_i}\right) \\
\frac{\partial m}{\partial t} &= \alpha_{\mathrm{m}}(V)(1-m) - \beta_{\mathrm{m}}(V) m \\
\frac{\partial h}{\partial t} &= \frac{h_\infty(V, [\mathrm{Ca}]_i) - h}{\tau_{h_{\mathrm{Ca(HVA)}}}} \\
\alpha_{\mathrm{m}}(V) &= 1.6/(1-\exp(-0.072(V-5))) \\
\beta_{\mathrm{m}}(V) &= -0.02(V+8.9)/(1-\exp((V+8.9)/5))
\end{aligned}
$$

$$
\begin{aligned}
h_\infty(V, [\mathrm{Ca}]_i) &= \left(\frac{2\ \mu M}{2\ \mu M + [\mathrm{Ca}]_i}\right)\frac{1}{(1+\exp((V+42)/8))} \\
\tau_{h_{\mathrm{Ca(HVA)}}} &= 0.02
\end{aligned}
$$

*Low-Voltage-Activated T-type calcium current* $I_{\mathrm{Ca(T)}}$

$$
\begin{aligned}
I_{\mathrm{Ca(T)}} &= \overline{g}_{\mathrm{Ca(T)}} m^2 h\ \mathrm{GHK}(V, [\mathrm{Ca}]_i, [\mathrm{Ca}]_o) \\
\mathrm{GHK}(V, [\mathrm{Ca}]_i, [\mathrm{Ca}]_o) &= 0.0853\frac{\mathcal{T}}{2}\left(\frac{\exp(2V\mathcal{F}/R\mathcal{T})[\mathrm{Ca}]_i/[\mathrm{Ca}]_o - 1}{(\exp(2V\mathcal{F}/R\mathcal{T}) - 1)}\right) \\
\frac{\partial m}{\partial t} &= \alpha_\mathrm{m}(V)(1-m) - \beta_\mathrm{m}(V)m \\
\frac{\partial h}{\partial t} &= \frac{h_\infty(V) - h}{\tau_{h_{\mathrm{Ca(T)}}}} \\
\alpha_\mathrm{m}(V) &= -0.2(V-19.26)/(\exp(-(V-19.26)/10)-1) \\
\beta_\mathrm{m}(V) &= 0.009\exp(-V/22.03) \\
\alpha_\mathrm{h}(V) &= 10^{-6}\exp(-V/16.26) \\
\beta_\mathrm{h}(V) &= 1/(\exp(-(V-29.79)/10)+1) \\
m_\infty(V) &= \alpha_\mathrm{m}(V)/(\alpha_\mathrm{m}(V)+\beta_\mathrm{m}(V)) \\
h_\infty(V) &= \alpha_\mathrm{h}(V)/(\alpha_\mathrm{h}(V)+\beta_\mathrm{h}(V)) \\
\tau_{m_{\mathrm{Ca(T)}}} &= 1/(\alpha_\mathrm{m}(V)+\beta_\mathrm{m}(V)) \\
\tau_{h_{\mathrm{Ca(T)}}} &= 1/(\alpha_\mathrm{h}(V)+\beta_\mathrm{h}(V))
\end{aligned}
$$

*Calcium gated potassium current* $I_{\mathrm{K(Ca)}}$

$$
\begin{aligned}
I_{\mathrm{K(Ca)}} &= \overline{g}_{\mathrm{K(Ca)}} m(E_{\mathrm{K(Ca)}} - V)\min([\mathrm{Ca}]_i/2\ (\mu M), 1) \\
\frac{\partial m}{\partial t} &= \alpha_\mathrm{m}(V)(1-m) - \beta_\mathrm{m}(V)m \\
\alpha_\mathrm{m}(V) &= 0.053\exp[(V+50)/11 - (V+53.5)/27] && \text{for } V \le 10 \\
&= 2\exp((-V-53.5)/27.5) && \text{for } V > 10 \\
\beta_\mathrm{m}(V) &= 2\exp((V+53.5)/27.5) - \alpha_\mathrm{m} && \text{for } V \le 10 \\
&= 0 && \text{for } V > 10
\end{aligned}
$$

*Muscarinic potassium current* $I_\mathrm{M}$

$$
\begin{aligned}
I_\mathrm{M} &= \overline{g}_{\mathrm{K_M}} n(E_{\mathrm{K_M}} - V) \\
\frac{\partial n}{\partial t} &= \frac{n_\infty(V) - n}{\tau_n(V)} \\
\alpha_\mathrm{n}(V) &= 0.001(V-30)/(1-\exp(-(V-30)/9)) \\
\beta_\mathrm{n}(V) &= -0.001(V-30)/(1-\exp((V-30)/9)) \\
n_\infty(V) &= \alpha_\mathrm{n}(v)/(\alpha_\mathrm{n}(V)+\beta_\mathrm{n}(V))
\end{aligned}
$$

$$\tau_{\mathrm{n_M}}(V) = 1/(\alpha_{\mathrm{n}}(V) + \beta_{\mathrm{n}}(V))$$

*Medium afterhyperpolarization current* $I_{\mathrm{mAHP}}$

$$\begin{aligned}
I_{\mathrm{mAHP}} &= \overline{g}_{\mathrm{mAHP}} m (E_{\mathrm{mAHP}} - V) \\
\frac{\partial m}{\partial t} &= \frac{m_\infty(V) - m}{\tau_{m_{\mathrm{mAHP}}}} \\
\alpha_{\mathrm{m}}(V) &= 0.48/(1 + 0.18/[\mathrm{Ca}]_i \exp(-1.68 V \mathcal{F}/RT) \\
\beta_{\mathrm{m}}(V) &= 0.28/\left(1 + \frac{[\mathrm{Ca}]_i}{0.011 \exp(-2V\mathcal{F}/RT)}\right) \\
m_\infty(V) &= \alpha_{\mathrm{m}}(V)/(\alpha_{\mathrm{m}}(V) + \beta_{\mathrm{m}}(V)) \\
\tau_{\mathrm{mAHP}}(V) &= 1/(\alpha_{\mathrm{m}}(V) + \beta_{\mathrm{m}}(V))
\end{aligned}$$

*Slow afterhyperpolarization current* $I_{\mathrm{sAHP}}$

$$\begin{aligned}
I_{\mathrm{sAHP}} &= \overline{g}_{\mathrm{sAHP}} m^3 (E_{\mathrm{sAHP}} - V) \\
\frac{dm}{dt} &= \frac{m_\infty([\mathrm{Ca}]_i) - m}{\tau_{\mathrm{sAHP}}([\mathrm{Ca}]_i)} \\
m_\infty([\mathrm{Ca}]_i) &= \frac{[\mathrm{Ca}]_i^2}{(0.025^2 + [\mathrm{Ca}]_i^2)} \\
\tau_{\mathrm{sAHP}}([\mathrm{Ca}]_i) &= \max\left(\frac{1}{(0.003\,(1 + ([\mathrm{Ca}]_i/0.025)^2))\,3^{(\mathcal{T}-251.16)/10}}, 0.5\right)
\end{aligned}$$

where $E_{\mathrm{leak}} = -80$ mV, $E_{\mathrm{Na}} = 50$ mV, $E_{\mathrm{Kdr}} = E_{\mathrm{K(A)}} = E_{\mathrm{K(Ca)}} = E_{\mathrm{mAHP}} = E_{\mathrm{sAHP}} = -90$ mV, and $E_{\mathrm{K(H)}} = -35$ mV, $\mathcal{F} = 96485$ C mol$^{-1}$ is Faraday's constant, $R = 8.1345$ J $^o$K$^{-1}$ mol$^{-1}$ is the gas constant, and $\mathcal{T}$ denotes absolute temperature in $^o$K (degrees kelvin). *Intracellular calcium dynamics* Calcium accumulation, extrusion, diffusion and buffering was simulated according to the following simple model which accounts for these processes by a simple exponential decay,

$$\frac{d[\mathrm{Ca}]_i}{dt} = \frac{[\mathrm{Ca}]_\infty - [\mathrm{Ca}]_i}{\tau_{\mathrm{Ca}}} + \frac{1}{2\mathcal{F}d}\left(I_{\mathrm{Ca(HVA)}} + I_{\mathrm{Ca(T)}} + I_{\mathrm{Ca(NMDA)}}\right)$$

where $[\mathrm{Ca}]_\infty = 20\mu M$ is the equilibrium concentration of intracellular calcium, $[\mathrm{Ca}]_i$ denotes the concentration of intracellular free calcium, $\tau_{\mathrm{Ca}} = 20$ msec is the diffusion rate constant, d is the depth of a shell just beneath the membrane, $I_{\mathrm{Ca(HVA)}}$ and $I_{\mathrm{Ca(T)}}$ respectively denotes the calcium current through L-type and T-type calcium channels, and $I_{\mathrm{Ca(NMDA)}} = 0.1 I_{\mathrm{NMDA}}$ is a fractional calcium current through postsynaptic NMDA receptors where 10% of the NMDA-mediated current is carried by calcium (**Garaschuk et al.**, 1996; **Burnashev et al.**, 1995).

The synaptic currents that are generated due to the incoming spike activity transmitted by impinging afferent fibers onto various locations across the dendrite result in depolaration and neuronal firing. The description of the four different synaptic currents generated by their respective underlying receptor types: AMPA, $\mathrm{GABA_A}$, $\mathrm{GABA_B}$, and NMDA, were modeled as follows,

**AMPA conductance and current:**

$$\begin{aligned} g_j^{\mathrm{AMPA}}(t) &= \overline{g}^{\mathrm{AMPA}} w_j(t) F \left( e^{-(t-t_i)/\tau_d^{AMPA}} - e^{-(t-t_i)/\tau_o^{AMPA}} \right) H(t-t_i), \\ I_j^{\mathrm{AMPA}}(t) &= g_j^{\mathrm{AMPA}}(t) \left( V - E_{\mathrm{rev}}^{\mathrm{AMPA}} \right) \end{aligned}$$

Note that only the synaptic efficacies or weights of $\mathrm{AMPA}$ currents generated at dendritic location $j$ are denoted by $w_j(t)$ and altered via STDP. All other weights (maximal conductances) associated with the other synaptic currents ($\mathrm{NMDA}$, $\mathrm{GABA_A}$, and $\mathrm{GABA_B}$) are fixed.

**$\mathrm{GABA_A}$ conductance and current:**

$$\begin{aligned} g_j^{\mathrm{GABA_A}}(t) &= \overline{g}^{\mathrm{GABA_A}} F \left( e^{-(t-t_i)/\tau_d^{GABA_\mathrm{A}}} - e^{-(t-t_i)/\tau_o^{GABA_\mathrm{A}}} \right) H(t-t_i), \\ I_j^{\mathrm{GABA_A}}(t) &= g_j^{\mathrm{GABA_A}}(t) \left( V - E_{\mathrm{rev}}^{\mathrm{GABA_A}} \right) \end{aligned}$$

where $E_{\mathrm{rev}}^{\mathrm{AMPA}} = 0$ mV, $E_{\mathrm{rev}}^{\mathrm{GABA_A}} = -80$ mV, $H(t)$ is the Heaviside step function, $w_j$ denotes the efficacy of the AMPA conductance in synapse $j$, and $F$ is a normalization factor such that an event with $\overline{g} = 1$ generates a peak conductance of 1 $\mu$S. The maximal AMPA $\overline{g}^{\mathrm{AMPA}}$ and $\mathrm{GABA_A}$ $\overline{g}^{\mathrm{GABA_A}}$ conductance were 5 and 2 nS, respectively. Onset and decay time constants were $\tau_o^{\mathrm{AMPA}} = 0.2$ msec and $\tau_d^{\mathrm{AMPA}} = 1.5$ msec for AMPA and $\tau_o^{\mathrm{GABA_A}} = 1.2$ msec and $\tau_d^{\mathrm{GABA_A}} = 9$ msec $\mathrm{GABA_A}$ conductances, respectively. Excitatory AMPA weights were initialized to $w_j(t) = 0.5$, but later changed by STDP.

**NMDA conductance and current:**

The postsynaptic NMDA conductance was modeled using a simple two state kinetic scheme represented by the following two state diagram

$$C + T \underset{\beta}{\overset{\alpha}{\rightleftharpoons}} O$$

where $\alpha$ and $\beta$ represent forward and backward voltage independent reaction rates. Defining $\zeta$ as the fraction of receptors in the open state of synapse $j$, then the above two state reaction is described by the following first order kinetic equation:

$$\frac{d\zeta_j}{dt} = \alpha\,[T]\,(1-\zeta_j) - \beta\zeta_j$$

where $\alpha = 10$ (msec)$^{-1}$ and $\beta = 0.0125$ (msec)$^{-1}$ denotes the forward binding and backward unbinding rates, respectively. The concentration [T] denotes a pulse of neurotransmitter of duration 1.1 msec in the synaptic cleft. The NMDA conductance is given by,

$$g_{\mathrm{j}}^{\mathrm{NMDA}}(t) = \overline{g}^{\mathrm{NMDA}} \zeta_{\mathrm{j}}(t)$$

while the NMDA current is,

$$I_{\mathrm{j}}^{\mathrm{NMDA}}(t) = g_{\mathrm{j}}^{\mathrm{NMDA}}(t)B(V)(V - E_{\mathrm{rev}}^{\mathrm{NMDA}})$$

where the reversal potential is $E_{\mathrm{rev}}^{\mathrm{NMDA}} = 0$, and $B(V)$ represents magnesium block described by the following voltage dependent process,

$$\mathrm{B}(V) = \frac{1}{1 + \exp(-0.062V)\left[\mathrm{Mg}^{2+}\right]_{\mathrm{o}}/3.57},$$

where the extracellular magnesium concentration was set to a value of $\left[\mathrm{Mg}^{2+}\right]_o = 1$ mM.

**$\mathbf{GABA_B}$ conductance and current:**

Post synaptic $\mathrm{GABA_B}$ receptor responses are activated by an intracellular second messenger system, mediated by fast G-protein binding to $K^+$ channels, whose state diagram is represented by the following kinetic scheme

$$\begin{aligned} R_o + T &\rightleftharpoons R \rightleftharpoons D \\ R + G_o &\rightleftharpoons RG \rightarrow R + G \\ G &\rightarrow G_o \\ C_1 + nG &\rightleftharpoons O \end{aligned}$$

The activated and desensitized forms of the receptor $G$ and $D$ respectively arise after neurotransmitter $T$ binds to the receptor $R_o$. Concurrently, the active form of the G-protein $G$ is produced after the inactive form $G_o$ has been catalyzed by $R$, and consequently binds to open $K^+$ channels, with $n = 4$ binding sites. The above kinetic scheme can be simplified, by assuming Michaelis-Menton kinetics, fast binding to $K^+$ channels, quasi stationarity of the intermediate enzymatic reactions, no receptor desensitization, and an excess of inactive G-proteins $G_o$, to the following system,

$$\begin{aligned} \frac{d\,[\mathrm{R_j}]}{dt} &= K_1\,[\mathrm{T}]\,(1 - [\mathrm{R_j}]) - K_2\,[\mathrm{R_j}] \\ \frac{d\,[\mathrm{G_j}]}{dt} &= K_3\,[\mathrm{T}]\,[\mathrm{R_j}] - K_4\,[\mathrm{G_j}] \\ g_j^{\mathrm{GABA_B}}(t) &= \bar{g}_j^{\mathrm{GABA_B}} \frac{[G(t)]^n}{[G(t)]^n + K_d} \\ I_j^{\mathrm{GABA_B}} &= g_j^{\mathrm{GABA_B}}(t)\left(V - E_{\mathrm{rev}}^{\mathrm{GABA_B}}\right) \end{aligned}$$

where $\bar{g}_j^{\mathrm{GABA_B}}$ is the maximal conductance of $\mathrm{GABA_B}$ receptors, $g_j^{\mathrm{GABA_B}}(t)$ denotes the $\mathrm{GABA_B}$ conductance, $K_d$ is the dissociation constant of G-protein binding to $K^+$ channels, and $E_{\mathrm{rev}}^{\mathrm{GABA_B}} = -95$ mV is the reversal potential.

## TABLE

**Table 1:** ***Ion Channel densities used in the Layer 2/3 pyramidal cell model***

| Symbol | Conductance density name | Density (pS/$\mu$m$^2$) | Reference(s) |
|---|---|---|---|
| $g_{\mathrm{L}}$ | Leak | 0.25; (With spines 0.3827) | (**Mainen et al.**, 1995) |
| $g_{\mathrm{Na}}$ | Sodium | 45 | (**Hamill et al.**, 1991; **Huguenard et al.**, 1988) |
| $g_{\mathrm{K}}$ | Potassium | 68 | (**Hamill et al.**, 1991) |
| $g_{\mathrm{K(A)}}$ | A-type potassium | 50 | (**Zhou and Hablitz**, 1996) |
| $g_{\mathrm{K(H)}}$ | Potassium H-current | Monotonically increasing | (**Traub et al.**, 2003) |
| $g_{\mathrm{Ca(HVA)}}$ | HVA L-type calcium | Monotonically increasing | (**Hamill et al.**, 1991) |
| $g_{\mathrm{Ca(T)}}$ | LVA T-type calcium | 4 | (**Hamill et al.**, 1991) |
| $g_{\mathrm{Ca(N)}}$ | N-type calcium | Monotonically increasing | (**Lazarewicz et al.**, 2002) |
| $g_{\mathrm{K(Ca)}}$ | $Ca^{2+}$-gated potassium | 40 | (**Kang et al.**, 1996) |
| $g_{\mathrm{K_M}}$ | M-type potassium | 3.5 | (**Rhodes and Gray**, 1994; **Rudolph and Destexhe**, 2003) |
| $g_{\mathrm{mAHP}}$ | Medium hyperpolarization | 15 | (**Rhodes and Gray**, 1994; **Poirazi et al.**, 2003) |
| $g_{\mathrm{sAHP}}$ | Slow hyperpolarization | 1.5 | (**Traub et al.**, 2003; **Poirazi et al.**, 2003) |

Stimulation was provided by a group of 250 inhibitory afferent fibers and two equally sized groups of 500 correlated excitatory afferents. Inhibitory and excitatory afferents are not correlated with each other. Furthermore, any afferent from one excitatory group was not correlated with any afferent from the other excitatory group. Put simply, the groups of correlated excitatory afferents were not correlated with each other (the correlation between any two different groups is zero). These groups of excitatory fibers will be referred to as groups A and B (when two groups are considered) and A,B,C, and D (when four groups are used). Each fiber, either excitatory or inhibitory, forms five random synaptic contacts in the model, as suggested by current anatomical data (**Markram et al.**, 1997; **Feldmeyer et al.**, 2002; **Thomson et al.**, 1994, 2002).

## REFERENCES

Burnashev, N., Zhou, Z., Neher, E., and Sakmann, B. (1995), Fractional calcium currents through recombinant GLUR channels of the NMDA, AMPA and kainate receptor subtypes., *J. Physiol.*, **485.2**, 403–418

Feldmeyer, D., Lubke, J., Silver, R. A., and Sakmann, B. (2002), Synaptic connections between layer 4 spiny neurone-layer 2/3 pyramidal cell pairs in juvenile rat barrel cortex: physiology and anatomy of interlaminar signalling within a cortical column, *J. Physiol.*, **538.3**, 803–822

Garaschuk, O., Schneggenburger, R., Schirra, C., Tempia, F., and Konnerth, A. (1996), Fractional CA2+ currents through somatic and dendritic glutamate receptor channels of rat hippocampal CA1 pyramidal neurones., *J. Physiol.*, **485.2**, 403–418

Hamill, O. P., Huguenard, J. R., and Prince, D. A. (1991), Patch-clamp studies of voltage-gated currents in identified neurons in the rat cerebral cortex, *Cereb. Cortex*, **1**, 48–61

Huguenard, J. R., Hamill, O. P., and Prince, D. A. (1988), Developmental changes in $Na^+$ condunctances in rat neocortical neurons: Appearance of a slowly inactivating component, *J. Neurophysiol.*, **59**, 778–795

Iannella, N. and Tanaka, S. (2006), Synaptic efficacy cluster formation across the dendrite via STDP, *Neurosci. Lett.*, **403**, 24–29

Iannella, N., Tuckwell, H., and Tanaka, S. (2004), Firing properties of a stochastic PDE model of a rat sensory cortex layer 2/3 pyramidal cell, *Math. Biosci.*, **188**, 117–132

Iannella, N. L., Launey, T., and Tanaka, S. (2010), Spike timing-dependent plasticity as the origin of the formation of clustered synaptic efficacy engrams., *Front Comput Neurosci*, 4

Kang, J., Huguenard, J. R., and Prince, D. A. (1996), Development of BK channels in neocortical pyramidal neurons, *J. Neurophysiol.*, **76**, 188–198

Lazarewicz, M. T., Migliore, M., and Ascoli, G. A. (2002), A new bursting model of ca3 pyramidal cell physiology suggests multiple locations for spike initiation, *Biosystems*, 67, 1, 129–137

Mainen, Z. F., Joerges, J., Huguenard, J. R., and Sejnowski, T. J. (1995), A model of spike initiation in neocortical pyramidal cells, *Neuron*, **15**, 1427–1439

Markram, H., Lubke, J., Frotscher, M., Roth, A., and Sakmann, B. (1997), Physiology and anatomy of synaptic connections between thick tufted pyramidal neurones in the developing rat neocortex, *J. Physiol.*, **500.2**, 409–440

Poirazi, P., Brannon, T., and Mel, B. W. (2003), Arithmetic of subthreshold synaptic summation in a model CA1 pyramidal cell, *Neuron*, **37**, 977–987

Rhodes, P. A. and Gray, C. M. (1994), Simulations of intrinsically bursting neocortical pyramidal neurons, *Neural Comput.*, **6**, 1086–1110

Rhodes, P. A. and Llinás, R. R. (2001), Apical tuft input efficacy in layer 5 pyramidal cells from rat visual cortex, *J. Physiol.*, **6**, 1086–1110

Rudolph, M. and Destexhe, A. (2003), A fast-conducting stochastic integrative mode for neocortical neurons *in vivo*, *J. Neurosci.*, **23**, 2466–2476

Thomson, A. M., Deuchars, J., and West, D. C. (1994), Relationships between morphology and physiology of pyramid-pyramid single axon connections in rat neocortex *in vitro*, *J. Physiol.*, **478.3**, 423–435

Thomson, A. M., West, D. C., Wang, Y., and Bannister, A. P. (2002), Synaptic connections and small circuits involving excitatory and inhibitory neurons in layers 2-5 of adult rat and cat neocortex:triple intracellular recordings and biocytin labelling *in vitro*, *Cereb. Cortex*, **12**, 936–953

Traub, R. D., Buhl, E. H., Gloveli, T., and Whittington, M. A. (2003), Fast rhythmic bursting can be induced in a layer 2/3 cortical neurons by enhancing persistent $Na^+$ conductance or by blocking bk channels, *J. Neurophysiol.*, **89**, 909–921

Zhou, F.-M. and Hablitz, J. J. (1996), Layer I neurons of rat neocortex. II. Voltage-dependent outward currents, *J. Neurophysiol.*, **76**, 668–682
